# Lignin-Derived Syringol and Acetosyringone from Palm Bunch Using Heterogeneous Oxidative Depolymerization over Mixed Metal Oxide Catalysts under Microwave Heating

**DOI:** 10.3390/molecules26247444

**Published:** 2021-12-08

**Authors:** Rangsalid Panyadee, Aphinan Saengsrichan, Pattaraporn Posoknistakul, Navadol Laosiripojana, Sakhon Ratchahat, Babasaheb M. Matsagar, Kevin C.-W. Wu, Chularat Sakdaronnarong

**Affiliations:** 1 Department of Chemical Engineering, Faculty of Engineering, Mahidol University, 999 Putthamonthon 4 Road, Salaya, Putthamonthon, Nakorn Pathom 73170, Thailand; rangsalid.p@gmail.com (R.P.); aphinan.sae@student.mahidol.edu (A.S.); pattaraporn.pos@mahidol.edu (P.P.); sakhon.rat@mahidol.edu (S.R.); 2 The Joint Graduate School of Energy and Environment (JGSEE), King Mongkut’s University of Technology Thonburi, 126 Pracha Uthit Road, Bang Mot, Tungkru, Bangkok 10140, Thailand; navadol@jgsee.kmutt.ac.th; 3 Department of Chemical Engineering, National Taiwan University, No.1, Sec. 4 Roosevelt Road, Taipei City 10617, Taiwan; bmatsagar22@gmail.com (B.M.M.); kevinwu@ntu.edu.tw (K.C.-W.W.); 4 Center of Atomic Initiative for New Materials (AI-MAT), National Taiwan University, Taipei City 10617, Taiwan; 5 International Graduate Program of Molecular Science and Technology, National Taiwan University (NTU), Taipei City 10617, Taiwan

**Keywords:** alkaline hydrothermal lignin fractionation, heterogeneous catalyst, phenolic compound, microwave-assisted lignin depolymerization, mixed iron and copper oxide catalyst

## Abstract

Biomass valorization to building block chemicals in food and pharmaceutical industries has tremendously gained attention. To produce monophenolic compounds from palm empty fruit bunch (EFB), EFB was subjected to alkaline hydrothermal extraction using NaOH or K_2_CO_3_ as a promotor. Subsequently, EFB-derived lignin was subjected to an oxidative depolymerization using Cu(II) and Fe(III) mixed metal oxides catalyst supported on γ-Al_2_O_3_ or SiO_2_ as the catalyst in the presence of hydrogen peroxide. The highest percentage of total phenolic compounds of 63.87 wt% was obtained from microwave-induced oxidative degradation of K_2_CO_3_ extracted lignin catalyzed by Cu-Fe/SiO_2_ catalyst. Main products from the aforementioned condition included 27.29 wt% of 2,4-di-tert-butylphenol, 19.21 wt% of syringol, 9.36 wt% of acetosyringone, 3.69 wt% of acetovanillone, 2.16 wt% of syringaldehyde, and 2.16 wt% of vanillin. Although the total phenolic compound from Cu-Fe/Al_2_O_3_ catalyst was lower (49.52 wt%) compared with that from Cu-Fe/SiO_2_ catalyst (63.87 wt%), Cu-Fe/Al_2_O_3_ catalyst provided the greater selectivity of main two value-added products, syringol and acetosyrigone, at 54.64% and 23.65%, respectively (78.29% total selectivity of two products) from the NaOH extracted lignin. The findings suggested a promising method for syringol and acetosyringone production from the oxidative heterogeneous lignin depolymerization under low power intensity microwave heating within a short reaction time of 30 min.

## 1. Introduction

To produce high-valued phenolic compounds from lignin, the researchers have proposed both thermochemical reactions, e.g., based-catalyzed/acid-catalyzed depolymerization [1,2], hydrogenation [3,4], hydrogenolysis [5,6], combustion [7], gasification [8], pyrolysis [9,10], and catalytic oxidation [11] approaches. In the past decades, hydrothermal reaction under high pressure and temperature has been proposed to produce either aromatic compounds or bio-oil from biomass [12,13]. In hydrothermal reactions, water was used as a reaction medium. At subcritical condition with high temperature and high pressure, water acts as catalyst behaving both basic and acidic properties. Apart from water, many other solvents could be used as the reaction medium to facilitate better reaction efficiency such as superior selectivity, higher reaction rate, and greater product yield. Moreover, both homogeneous and heterogeneous catalysts could be used to improve the reaction performance [14]. Advantages of hydrothermal technique were the higher yield of phenolic compounds as well as economical and simple handling. Recently, Chan et al. (2015) studied the process parameters for the hydrothermal liquefaction of waste from the palm oil industry for phenolic bio-oil production [15]. The proposed technology although provides high phenolic compound yield, a great amount of energy is required as the temperature range of hydrothermal liquefaction over 350 °C is applied. In addition, the high capital expenditure due to the high-pressure vessel beyond 8 MPa is needed depending on the solvents used in the reaction. Apart from that, a previous research reported successful vanillin production under thermal condition (400–600 °C) that required special reactor having capability to control reaction time down to 40–600 s [16]. Therefore, two-step lignin fractionation followed by lignin depolymerization under mild hydrothermal reaction in alkaline condition has been proposed in the present work.

In case of lignin depolymerization to phenolic compounds, there were five types of reactions commonly used, consisting of metallic-catalyzed, base-catalyzed, acid-catalyzed, ionic liquids (ILs)-induced, and supercritical solvolysis lignin depolymerization reactions. It was found that vanillin was successfully produced from dissolution of kraft lignin and eucalyptus via ILs pretreatment at 160 °C for 6 h while syringol and allyl guaiacol were the major products observed from dissolution of switch grass and pine, respectively [17]. Various ILs assisted lignin depolymerization processes with high selectivity were also proposed [18,19,20], but the ILs cost and recyclability are limitations. Ordinarily, base-catalyzed and acid-catalyzed depolymerization reaction were conscientious, but low selectivity was obtained. Not only the strong reaction conditions (high temperature, high pressure and high pH) but also requirement of extraordinarily designed reactors, resulted in high costs of phenolics production. Further, supercritical fluids although provides high selectivity than acid and base-catalyzed reactions, nevertheless supercritical solvents facility limited their applications on biomass treatment in commercial scale [21,22]. Conversion of lignin to vanillin or phenolic aldehydes e.g., *p*-hydroxybenzaldehyde, vanillaldehyde, syringaldehyde [23], which are used in pharmaceutical application, has been widely studied via mild oxidative reaction that required either air, molecular oxygen [24] or oxidant such as H_2_O_2_ [25,26,27].

Additionally, metal-catalyzed oxidative lignin depolymerization has offered great advantages because of its high selectivity and relatively milder reaction condition; therefore, metal supported catalysts have been extensively used for lignin valorization [13,28,29]. It has been reported that Au/TiO_2_, however, favored ring-opening reactions of lignin while Pt/TiO_2_ effectively promoted lignin condensation and gave minimal effect on ring-opening reaction [30]. Although precious metal-supported catalysts are efficient for the valorization of lignin, their utilization is not economically feasible because of limited availability and high cost. To avoid these issues, non-precious metal supported catalysts have been introduced for the efficient heterogeneous lignin depolymerization. Among all metal complex investigated, the copper complexes could influence the mechanism in accordance with formation of monophenolic compounds. It was revealed that the Cu and La-doped porous metal oxide-based catalysts derived from hydrotalcite-like precursors were promising catalysts for the depolymerization of organosolv lignin in supercritical methanol [31]. In this method, lignin was depolymerized to methanol-soluble products without any char formation. The obtained bio-oil contains oligomers with high aromatic content and phenolic monomers. Most of early research on lignin oxidation was proceeded with oxidant or with Zr^4+^, Mn^3+^, Co^2+^ and Cu^2+^ which were simple transition metal ions [32,33]. After that, Mn, Co, Cu and Fe based metal oxides (e.g., CuO, MnO_2_), metal chlorides (e.g., MnCl_2_, CoCl_2_, FeCl_3_) [26,34] and composite metal oxides were subsequently investigated to augment oxygen catalytic efficiency for lignin depolymerization [35,36,37,38].

Recently, lignin depolymerization using microwave heating has been widely investigated due to its high heating rate and more selective to break down particular bonding thus yielding high selectivity of desire products based on individual catalyst compared with conventional heating approaches [4,39,40]. Liu and colleagues newly reported on lignin degradation in isopropanol with very high liquid yield at 45.35 wt% within only 30 min under microwave heating at 120 °C [39]. Even higher liquid product yield at 72.0 wt% including 6.7 wt% monomers, mainly 2,3-dihydrobenzofuran (3.00 wt%) and p-coumaric acid (1.59 wt%), from alkaline lignin depolymerization at 160 °C in formic acid/methanol media were achieved within 30 min [40]. A study just newly revealed the catalytic C-O-C bond scission of birch sawdust lignin promoted by Fe(OTf)_3_ under the identical conditions (190 °C, 1 h), which yielded more selective syringyl unit (S) of lignin monomer compared with guaiacyl-unit (G) of lignin [41]. Similar result of C-O-C ether bond cleavage was found when Rh/C was the catalyst and formic acid was used as the reaction medium under microwave heating [13]. Just newly reported, microwave-assisted catalytic depolymerization of birch sawdust lignin over Pt/C, Pd/C, or Ru/C in water/alcohol mixture facilitated in situ hydrogen generated and simultaneously promoted the hydrogenolysis of β-O-4 ether linkage which markedly yield S-type lignin relatively to Guaiacyl or G-type lignin as main products [42]. The result was in good agreement with our previous study on microwave-assisted depolymerization of alkaline lignin from palm bunch over dual Cu(OH)_2_ and Fe_2_O_3_ catalysts which gave highly selective syringyl-type products within only 15 min [26].

In the present work, based on our previous study Fe and Cu exhibited very good performance on lignin depolymerization under mild microwave heating in the presence of H_2_O_2_ in homogeneous catalytic system [26]. A high yield of oxidative lignin depolymerization products, namely, syringol, acetosyringone and vanillin, were produced with high selectivity. Therefore, heterogeneous Fe and Cu based mixed metal oxide catalysts were synthesized on various supports and used as the catalysts for the depolymerization of the EFB derived alkaline lignin to produce monophenolic compounds. To the best of our knowledge, there was no report on investigation of mixed metal oxide Fe_2_O_3_/CuO/SiO_2_ and Fe_2_O_3_/CuO/Al_2_O_3_ used as catalyst in oxidative lignin depolymerization. Therefore, heterogeneously mixed metal oxide (Fe_2_O_3_ and CuO) catalysts were synthesized on different supports (SiO_2_ or Al_2_O_3_) and their catalytic activity under oxidative condition using microwave heating were compared. The synthesized catalyst was easily recovered by filtration or centrifugation that is beneficial for recycling the catalyst. The results from homogeneous catalytic lignin depolymerization and heterogeneous catalytic reaction were compared.

## 2. Materials and Methods

### 2.1. Biomass and Chemicals

To prepare the material for lignin extraction, raw EFB from a palm oil mill having initial moisture content at ~50% was washed with water and sun-dried for 12 h. After that, it was dried at 80 °C in an oven for 24 h to obtain 4.3% final moisture content. Then, dried EFB was crushed and sieved to the particle size in a range of +50/−200 mesh (74–297 μm), and stored in a desiccator for use. For catalyst synthesis, silicon dioxide (SiO_2_) and aluminium oxide (Al_2_O_3_) were purchased from KemAus, Australia and used as the catalyst support. Copper (II) nitrate (Cu(NO_3_)_2_) and iron (III) nitrate (Fe(NO_3_)_3_) were obtained from Ajax Finechem, Australia. For lignin separation from EFB, the chemicals namely potassium carbonate (99.8%, Daejung, Siheung-si, Korea), sodium hydroxide (99.8%, Ajax Finechem, New South Wales, Australia), hydrogen peroxide (30% *w*/*w*, Ajax), sulfuric acid (98%, RCI Labscan, Bangkok, Thailand), and hydrochloric acid (37%, RCI Labscan) were purchased and used as received. Solvents for phenolic compound extraction and GC-MS analysis such as methanol (99.8%, HPLC, RCI Labscan) and ethyl acetate (99.5%, Daejung) were acquired and used as received.

### 2.2. Co-Impregnation of SiO_2_, Al_2_O_3_ Supported Cu-Fe Catalysts for EFB-Extracted Lignin Depolymerization

**Figure 1:**
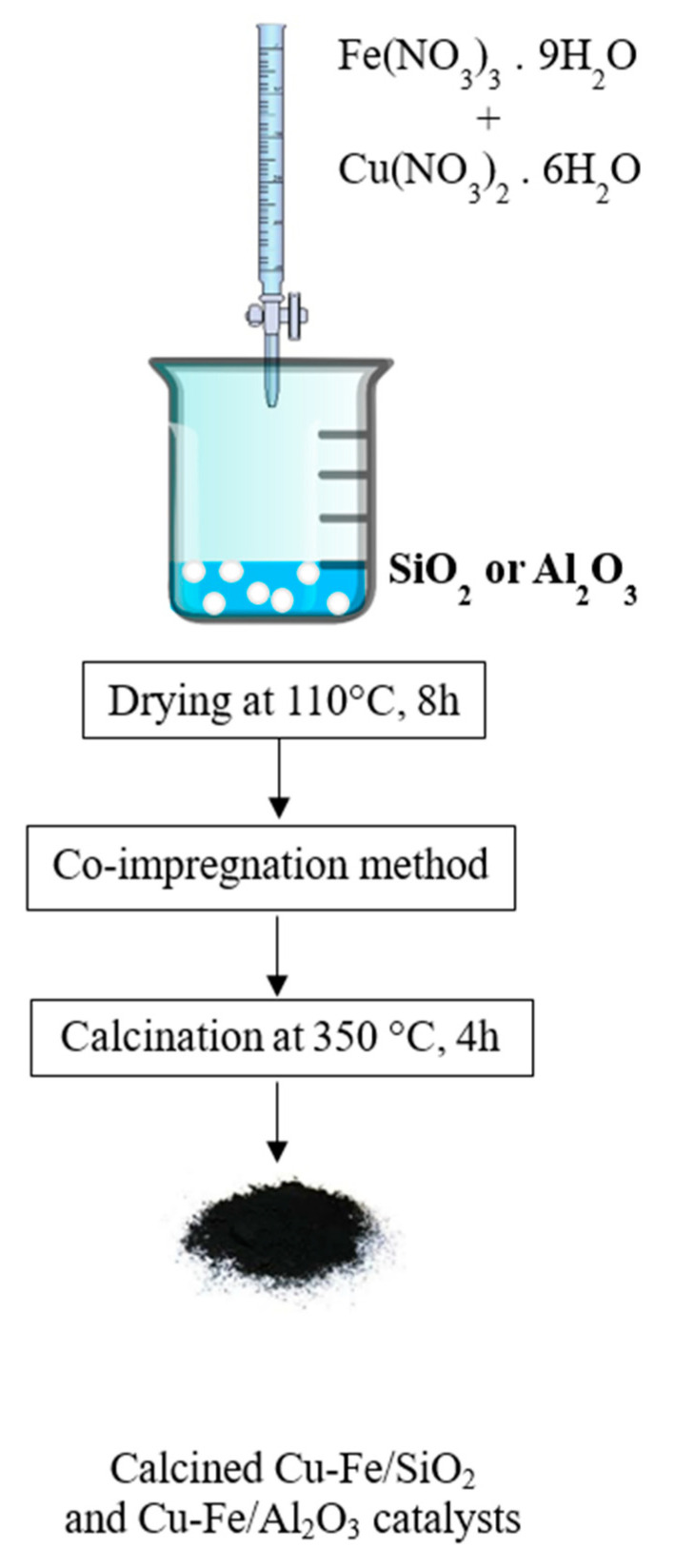
Co-impregnation of Cu-Fe catalysts on SiO_2_ or Al_2_O_3_ supports.

Both the Cu and Fe loadings of the catalysts were 10 mol% based on SiO_2_ and Al_2_O_3_. The aqueous mixture solution of Cu(NO_3_)_2_ and Fe(NO_3_)_3_ were prepared and added dropwise in the SiO_2_ or Al_2_O_3_ in a crucible. The slurry was evaporated in ambient atmosphere for 8 h, then dried at 110 °C overnight, and calcined in furnace at 350 °C in an excess air for 4 h, as shown in Figure 1. The calcined Cu-Fe/Al_2_O_3_ and Cu-Fe/SiO_2_ catalysts were stored in an automatic desiccator at <25% relative humidity.

### 2.3. Heterogeneously Mixed Metal Oxides Complex Catalysts Characterization

The crystal structure of heterogeneously mixed metal oxides catalysts was characterized by X-ray diffractometry (XRD, D8 Advance, Bruker, Bremen, Germany) with scan rate at 1° min^−1^ and 2ϴ range from 10° to 70°. The surface elemental composition of the calcined catalysts was determined by X-ray photoelectron spectroscopy (XPS, AXIS Nova, Kratos, Manchester, UK). Quasi-quantitative analysis of metal oxides in calcined catalysts was performed using X-ray Fluorescence Spectrometer (XRF, model Rigaku ZSK Primus, Rigaku, Tokyo, Japan). The appearance and elemental composition of catalysts were analyzed by Scanning electron microscopy with energy-dispersive X-ray spectroscopy (SEM-EDX) (VEGA3, TESCAN Brno-Kohoutovice, Czech Republic). Field Emission Scanning Electron Microscope (FE-SEM) model JEOL JSM7800F, JAPAN, Software: PCSEM equipped with Energy Dispersive X-ray Spectrometer (EDS) model Oxford X-Max 20, United Kingdom (UK) was used for analysis of elemental dispersion on catalyst surface with accelerating voltage of 15 kV at 2500–5000 magnification. Analysis of ammonia-temperature programmed desorption (NH_3_-TPD) using chemisorption analyzer (BEL Japan Inc.) was applied to quantify the acid density and the distribution of acid sites of synthesized catalysts and the support in a temperature range of 100 and 700 °C.

### 2.4. Lignin Extraction and Depolymerization of Lignin

#### 2.4.1. Alkali Hydrothermal Extraction of Lignin from Palm Empty Fruit Bunch

Lignin extraction from EFB was described in our previous study [26]. First, dried EFB was crushed to small particles and sieved to a range of +50/−200 mesh. Then, lignin fractionation from EFB using alkaline solution (1 mol L^−1^ K_2_CO_3_ or NaOH solution) was conducted in a high-pressure stainless-steel hydrothermal reactor with solid-to-liquid ratio of 1:5. The reaction was performed at 200 °C for 20 min under 2 MPa nitrogen pressure. For lignin precipitation, lignin-rich solution from alkali hydrothermal extraction was acidified with concentrated sulfuric acid until final pH of solution was 1.0. The solid precipitate was separated from solution by centrifuge at 7000 rpm at 25 °C for 15 min. Then, solid precipitated lignin was washed with distilled water until the pH became neutral. Finally, alkaline extracted lignin was dried at 50 °C for 18 h and used as the precursor for the production of phenolic compounds.

#### 2.4.2. Microwave-Assisted Phenolic Compound Production over Heterogeneously Mixed Metal Oxides Complex Catalyst

**Figure 2:**
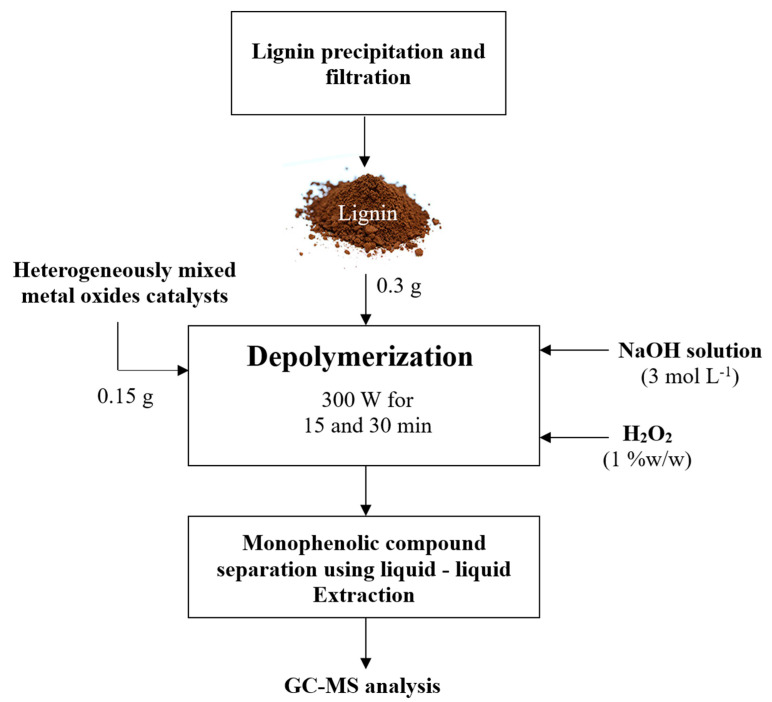
Experimental diagram of EFB depolymerization process in a microwave reactor with mixed metal oxides complex heterogeneous catalysts.

The reaction catalyzed by Cu(OH)_2_ + Fe_2_O_3_ mixed metal oxides catalyst with 1 wt% and 2.5 wt% H_2_O_2_ was selected as it was the best condition for homogeneous monophenolic compound production from K_2_CO_3_-lignin and NaOH-lignin, respectively. Based on our previous study [26], the reaction was carried out under microwave irradiation at 300 W for 15 and 30 min for 0.3 g K_2_CO_3_-lignin or NaOH-lignin with 0.15 g of heterogeneously mixed metal oxide catalyst and 1 wt% of H_2_O_2_ as an oxidant in the presence of 3 mol L^−1^ NaOH solution as demonstrated in Figure 2.

Recyclability of both CuFe/Al_2_O_3_ and CuFe/SiO_2_ catalysts on NaOH-lignin in microwave depolymerization at 300 W for 30 min was studied. Spent catalysts after the first reaction was filtered and washed several times with methanol to eliminate lignin contamination. Dry catalysts at 60 °C for 12 h were used for the subsequent reaction with the same weight ratio of catalyst to lignin when solid-to-liquid ratio was constant for all catalyst recycle studies. Spent catalysts were characterized using XPS for elemental analysis compared with fresh catalyst.

### 2.5. Analysis of Lignin Functional Groups and Lignin Depolymerization Products

Analysis of K_2_CO_3_-lignin and NaOH-lignin was performed after acid precipitation of lignin from alkali hydrothermal extraction using sulfuric acid, pH 1.0. The precipitate was centrifuged and dried at 50 °C for 18 h. Fourier transform infrared (FT-IR) spectroscopy (Nicolet 6700, Thermo Fisher Scientific, Waltham, MA, USA) was used to analyze functional groups of extracted lignin at the wavenumber ranging from 4000 to 400 cm^−1^ with 4 cm^−1^ resolution and 100 scan numbers. In order to identify and compare the different amounts of functional groups, 0.01 g lignin sample was mixed with 0.99 g KBr for palletization prior to FT-IR spectroscopy. In case of analysis of lignin depolymerization product from microwave reaction, ethyl acetate extraction of monophenolic compounds from the liquid products from depolymerization reaction was conducted, subsequently the solvent was evaporated under vacuum, and the dry product was re-dissolved in methanol for gas chromatography mass spectrometry (GC-MS) analysis (Agilent GC6890N, Wilmington, DE, USA). The extracts dissolved in methanol (1 μL) was injected into the capillary HP-5 MS column (30 m × 0.25 mm × 0. 25 μm) controlled at 250 °C using splitless mode. Helium was used as a carrier gas with a flow rate of 1 mL min^−1^. In case of product quantification, known concentration of main products in the reaction mixture (e.g., syringol, vanillin, acetosyringol, acetovanillone, syringaldehyde, and 2,4-di-tert-butylphenol) was analyzed by gas chromatography-flame ionization detector (GC-FID, model Clarus 580, Perkin Elmer, Waltham, MA, USA).

## 3. Results and Discussion

### 3.1. Extracted Lignin from EFB

**Figure 3:**
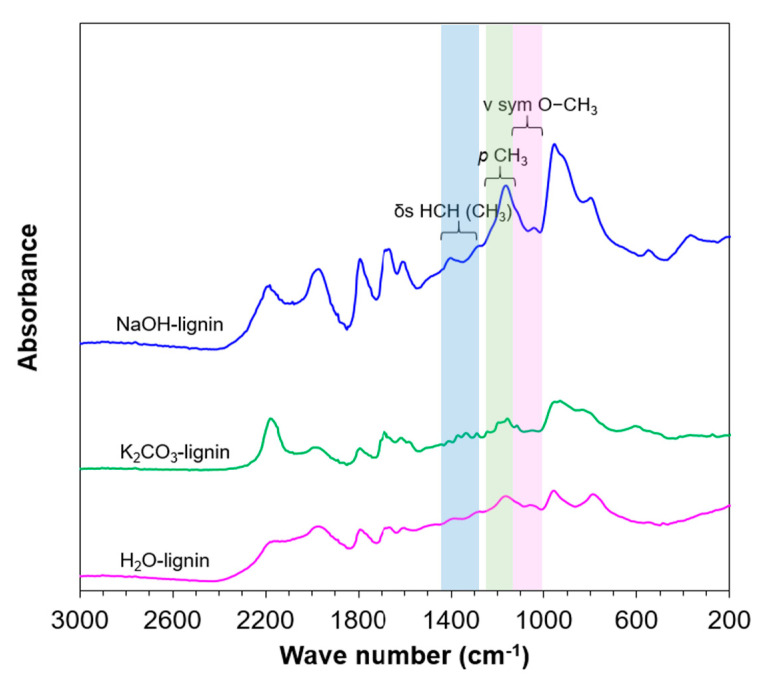
FT-IR spectroscopic analysis of NaOH-lignin and K_2_CO_3_–lignin compared with the control (H_2_O-lignin) from the alkali hydrothermal lignin extraction with solid:liquid ratio of 1:5 at 200 °C for 20 min under 2 MPa nitrogen pressure.

The properties of extracted lignin from EFB using K_2_CO_3_ and NaOH solution in hydrothermal reactor were reported elsewhere [26]. As shown in Figure 3, FT-IR spectra of NaOH-lignin and K_2_CO_3_-lignin were noticeably different especially methyl (CH_3_) intensity compared with the control when lignin was hydrothermally extracted without alkali. FT-IR peaks could be used to identify the presence of CH_3_ group in extracted lignin indicating by peak intensity at wave number of 1028–1052 cm^−1^ (symmetry O–CH_3_ vibration), ~1176 cm^−1^ (ρ CH_3_) and 1442–1463 cm^−1^ (δ_s_ HCH (CH_3_)) [43]. It was observed that methyl content in extracted lignin using different extractants was found in a respective degree; NaOH-lignin > H_2_O-lignin > K_2_CO_3_-lignin (Figure 3). NaOH-lignin was found to contain the highest concentration of CH_3_ group. It was reported that hydroxide ions assist β-O-4 ether bonds cleavage by acting as a nucleophile. Na+ ions adducted with lignin molecules could polarize the ether bonds rendering an enhancement of negative charge of oxygen atom of the ether bond and thus the energy for heterolytic breakdown of the linkage is decreased [44]. After delignification and alkaline degradation, the obtained alkali lignin consists mainly of three phenyl-propane units. The reactive sites for heterogeneously catalytic conversion to phenolic compounds i.e., hydroxyl, methoxyl, and aldehyde groups were increased [45].

In contrast, alkali carbonates (i.e., K_2_CO_3_) were determined to influence a decrease of proton concentration during depolymerization reaction and led to enhancing parallel and secondary reaction mechanism to generate more phenols and conjugated phenolic compounds from demethylation of original lignin [46]. From the K_2_CO_3_ extraction condition, the smaller molecular weight lignin was obtained relative to NaOH-lignin from gel permeation chromatography (GPC) due to greater amount of basic ions i.e., K^+^ and CO_3_^2-^ compared with Na^+^ and OH^−^ at the similar molar concentration (1 mol L^−1^) [26]. K_2_CO_3_-lignin has smaller molecular weight of 1125 g mol^−1^ but lower polydispersity index (PD) of 1.53 when compared with NaOH-lignin that yielded 1244 g mol^−1^ molecular weight with greater PD of 1.58. These smaller K_2_CO_3_ extracted lignin molecules possibly tended to be more effortless to depolymerize to monophenolic products using heterogeneously mixed metal oxide catalyst and hydrogen peroxide in the following section.

### 3.2. Characterization and Reactivity of the Heterogeneously Mixed Metal Oxides Catalysts on Phenolic Compounds Production

#### 3.2.1. X-ray Diffraction (XRD) and X-ray Fluorescence Spectrometry (XRF) of Heterogeneously Mixed Metal Oxide Catalysts

**Figure 4:**
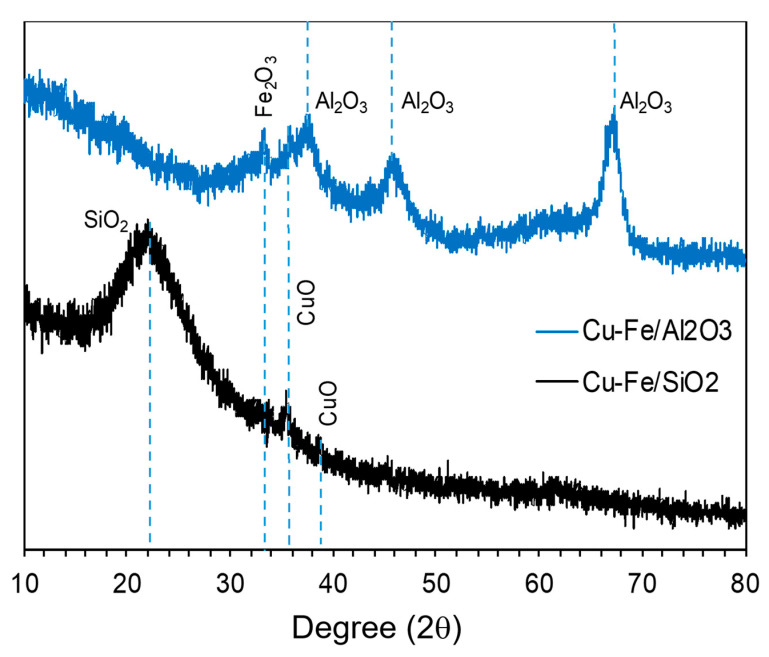
XRD patterns of heterogeneously Cu-Fe/SiO_2_ and Cu-Fe/Al_2_O_3_ mixed metal oxides catalysts.

As demonstrated in Figure 4, the XRD patterns of Cu-Fe/Al_2_O_3_ and Cu-Fe/SiO_2_ catalysts show diffraction peaks at 2θ = 35.4° and 39.4° corresponding to CuO. Small peak attributable to CuO was observed, suggesting that Cu was present as amorphous or highly dispersed form on the support [47]. The peak at 33.4° ascribed to the presence of Fe_2_O_3_ [48] were active phases for the lignin depolymerization reaction. A very broad peak at 2θ of 22.4° observed on the catalyst was attributed to amorphous SiO_2_ and the peaks at 2θ = 37.6°, 46.1°, and 67° were ascribed to the Al_2_O_3_ support (Figure 4).

The quantitative analysis of metal oxides in synthesized catalysts by XRF technique was also reported in Table 1. After calcination at 350 °C for 4 h under excess air, Cu:Fe molar ratio of 1:1 from both Cu-Fe/Al_2_O_3_ and Cu-Fe/SiO_2_ catalysts remained the same amount as precursor prepared. The results exhibited that the percentages of metal oxides in Cu-Fe/Al_2_O_3_ catalyst were 12.80% CuO, 8.15% Fe_2_O_3_, 78.67% Al_2_O_3_ and 0.07% SiO_2_ by weight, while Cu-Fe/SiO_2_ catalyst contained 12.27% CuO, 10.38% Fe_2_O_3_, 0.12% Al_2_O_3_ and 76.36% SiO_2_. Majority of metal oxides from Cu and Fe was CuO or Cu^2+^ and Fe_2_O_3_ or Fe^3+^ while Al_2_O_3_ and SiO_2_ support remained the same phase as initial form. The XRF results of all catalysts and supports were corresponded with XRD pattern from Figure 4.

#### 3.2.2. X-ray Photoelectron Spectroscopy (XPS) of Heterogeneously Mixed Metal Oxides Catalysts

**Figure 5:**
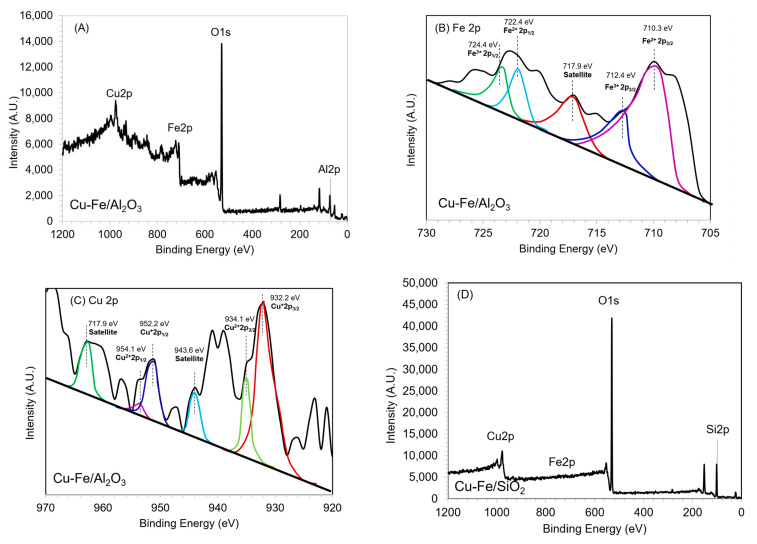

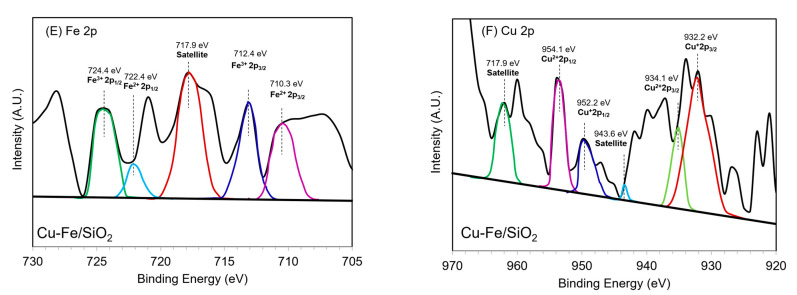
XPS spectra recorded for (**A**) overall spectrum, (**B**) Fe 2p, (**C**) Cu 2p of Cu-Fe/Al_2_O_3_ catalyst, and (**D**) overall spectrum, (**E**) Fe 2p and (**F**) Cu 2p of Cu-Fe/SiO_2_ catalyst.

To understand more insights into the oxidation state of Fe and Cu species in synthesized mixed metal oxide catalyst, the overall XPS analysis of Cu and Fe on Al_2_O_3_ and SiO_2_ support was performed as shown in Figure 5A,D. Chemical surface state of catalysts contained majority of O 1s, Cu 2p, and Fe 2p for the active species as well as Al 2p and Si 2p for the support according to the precursors. For Cu-Fe/Al_2_O_3_ catalyst, Fe 2p_1/2_ and Fe 2p_3/2_ spinning orbit peaks were illustrated in Figure 5B. The Fe 2p_3/2_ peaks represented Fe^3+^ and Fe^2+^ species were detected at binding energy of 712.4 and 710.3 eV attributed to the presence of Fe_2_O_3_ and FeO, respectively, while the satellite vibration peak of Fe was observed at 717.9 eV [49,50]. The peak intensity in XPS analysis suggested that the binding energy of FeO was slightly lower than Fe_2_O_3_, and the oxidized FeO could generate Fe_2_O_3_ during calcination process in excess of air.

In case of copper species, the XPS spectra showed the predominantly spinning orbit peaks for Cu 2p_3/2_ and Cu 2p_1/2_ corresponding to the binding energy values at 934 and 954.1 eV, respectively. This was in good concordance with the result in previous literature [51,52,53]. Cu 2p_3/2_ XPS peaks of Cu^2+^ and Cu^+^ species indicating the presence of CuO and Cu_2_O after calcination process were prominent at binding energy of 934.1 and 932.2 eV, respectively (Figure 5C). CuO/Cu_2_O oxygen carriers are the higher oxygen transport capacity and higher reactivity [54], thus it is suitable for facilitating oxidative depolymerization of lignin. The shake-up satellite peak of Cu at 943.6 eV was observed which was well corresponded to a previous work [55]. Moreover, the down shifted XPS peak from 934 to 932 eV referred to the Cu^2+^ ion on catalyst surface concentration while metallic Cu^0^ was not obviously detected in Cu-Fe/Al_2_O_3_ and Cu-Fe/SiO_2_ catalysts. It has also been observed that Cu oxides do not react with the SiO_2_ and have the high reactivity and oxygen transport capacity [56]. The oxidation state and electron vacancy of Fe and Cu on catalyst surface substantially influences the catalytic pathway of lignin depolymerization to phenolic compounds. Similar results were found for Cu-Fe/SiO_2_ (Figure 5D–F); however, when compared with Al_2_O_3_ support, Fe^2+^ species attributed to FeO were less intense compared to Fe^3+^ assigned to Fe_2_O_3_. This was confirmed by XRF results demonstrated in Table 1. Since the oxidation state of iron species is Fe_1−x_O→Fe_3_O_4_→Fe_2_O_3_ [57], the depletion of oxygen during calcination from the trade-off between copper and iron species possibly causes the presence of mixed FeO/Fe_2_O_3_ and Cu_2_O/CuO as shown in XPS peaks. This occurrence may facilitate the greater acid state of Cu-Fe/Al_2_O_3_ and more basic state of Cu-Fe/SiO_2_ which could be characterized by NH_3_-TPD analysis.

#### 3.2.3. NH_3_-TPD Analysis of Synthesized Catalysts

Variation of temperature from low to high levels in NH_3_ adsorption-desorption process was performed to analyze the strength of acidity in the synthesized catalyst. As illustrated in Figure S1, the peak appeared in the temperature range from 150 °C to 200 °C found in Cu-Fe/Al_2_O_3_, Al_2_O_3_, Cu-Fe/SiO_2_ and SiO_2_ indicated the weak acid sites or weak interaction of ammonia with copper and iron oxides as well as the Al_2_O_3_ and SiO_2_ supports. This peak at low temperature was ascribed to weakly bound ammonia onto the catalysts whereas the peak at higher temperature corresponds to ammonia specifically adsorbed onto the acid sites. It has been previously reported that very strong acid sites (h^+^-peak) were found between 550 °C to 700 °C [58] which were considerably found in Cu-Fe/SiO_2_, and SiO_2_ indicating very strong acid sites in the catalysts.

For NH_3_-TPD analysis, the peak position gives information about the relative acid strength while the width of the peak provides evidence of the distribution of the strength under identical experimental conditions. To calculate the binding strength of the acid sites, a theoretical model is an effective tool when slow diffusion as the rate-limiting step has to be excluded [59,60] and the total acid sites could be quantified by the integration of peak area from NH_3_-TPD chromatograms. As shown in Table S1, the total acid site density of synthesized catalysts and the supports was calculated based on the absorption and desorption of ammonia when the temperature range was 100 and 700 °C (Figure S1). Comparing at the same dry weight of materials, the addition of metal oxides, Cu(NO_3_)_2_ and (Fe(NO_3_)_3_) as precursors, by doping into the Al_2_O_3_ and SiO_2_ supports significantly decreased the acid site density as shown in Table S1.

#### 3.2.4. Field Emission Scanning Electron Microscopy with Energy-Dispersive X-ray Spectroscopy (FESEM-EDX) Mapping of Heterogeneously Mixed Metal Oxides Catalysts

**Figure 6:**
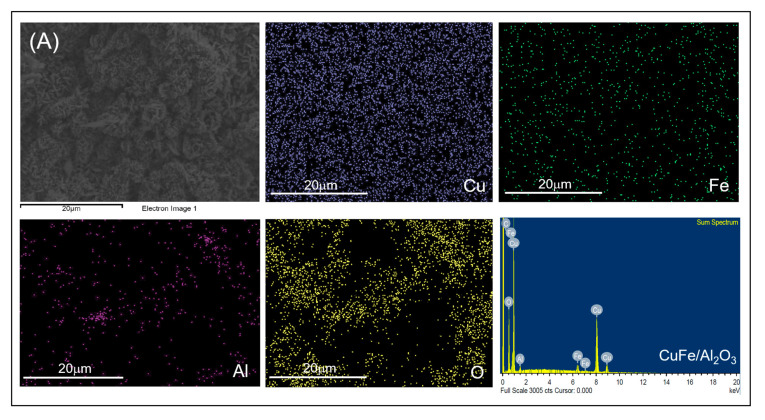

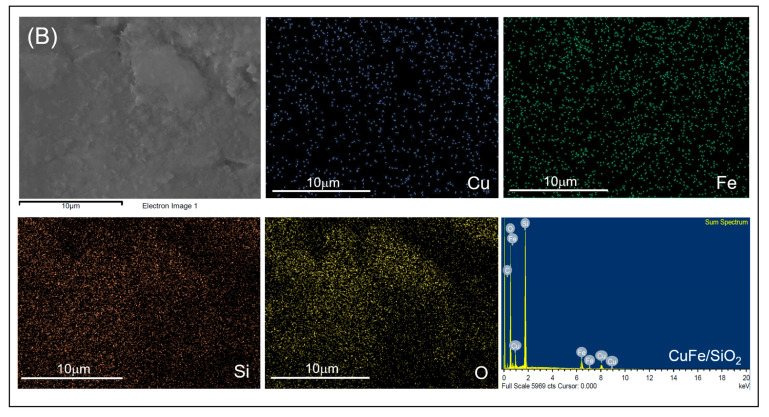
FESEM-EDX elemental mapping of (**A**) Cu-Fe/Al_2_O_3_ and (**B**) Cu-Fe/SiO_2_ catalysts.

The morphological and surface elemental composition of heterogeneously mixed metal oxides Cu-Fe/SiO_2_ and Cu-Fe/Al_2_O_3_ catalysts were analyzed with field emission scanning electron microscopy with energy dispersive X-ray spectroscopy (FESEM-EDX) as illustrated in Figure 6 and Figures S2 and S3. The EDX mapping analysis showed the similar pattern of Cu and Fe ions from co-impregnation that were well dispersed on Al_2_O_3_ and SiO_2_ supports. The surface elemental analysis results showed the presence of Cu and Fe on Al_2_O_3_ and SiO_2_ support accordingly as demonstrated in Tables S2 and S3, respectively. Therefore, the co-impregnation technique for mixed metal oxides catalyst synthesis was suitable to form the metal oxide catalysts on the support without either agglomeration or growth of metal crystal cluster. The morphology of the synthesized Cu-Fe/Al_2_O_3_ and Cu-Fe/SiO_2_ catalysts after Cu and Fe impregnation was analyzed by scanning electron microscopic (SEM) technique as illustrated in Figure S4. The SiO_2_ support was the finest particle with 1000 magnification and having 5–20 µm in particle size.

### 3.3. Phenolic Compounds Production from K_2_CO_3_-Lignin and NaOH-Lignin with Heterogeneously Mixed Metal Oxides Catalysts

After the synthesis of heterogeneously mixed metal oxides catalysts, they were used for microwave-assisted hydrothermal depolymerization of K_2_CO_3_-lignin and NaOH-lignin to produce phenolic compounds. From the previous experiment, the optimal condition for homogeneous lignin depolymerization to specific products was the microwave-assisted reaction catalyzed by Cu(OH)_2_ + Fe_2_O_3_ co-catalyst at 300 W for 15 and 30 min with 1 wt% of H_2_O_2_ [26]. Thus, for the present experiment on heterogeneous lignin depolymerization using mixed metal oxides catalyst, the aforementioned optimal condition was selected and the reaction took place for 15 and 30 min for both K_2_CO_3_-lignin and NaOH-lignin.

From the GC-MS analysis, the percentage of phenolic compound concentration was summarized in Table 2. The highest percentage of total phenolic compound concentration of 63.87 wt% was obtained from microwave-assisted oxidative degradation of K_2_CO_3_-lignin when the lignin degradation reaction was at 300 W, 30 min with 1.0 wt% H_2_O_2_ and catalyzed by Cu-Fe/SiO_2_ catalyst. The main products from aforementioned condition contained 19.21 wt% of syringol, 2.16 wt% of vanillin, 3.69 wt% of acetovanillone, 2.16 wt% of syringaldehyde, 9.36 wt% of acetosyringone and 27.29 wt% of 2,4-di-tert-butylphenol (Figures S5 and S6). In case of NaOH-lignin, the highest percentage of phenolic compound concentration was 49.52 wt%. The major products included 27.06 wt% of syringol, 1.61 wt% of vanillin, 4.39 wt% of acetovanillone, 1.97 wt% of syringaldehyde, 11.71 wt% of acetosyringone and 13.09 wt% of 2,4-di-tert-butylphenol when the lignin depolymerization reaction was conducted with 1.0 wt% H_2_O_2_ and Cu-Fe/Al_2_O_3_ catalyst for 30 min (Figures S7 and S8). Although Cu-Fe/SiO_2_ catalyzed the K_2_CO_3_-lignin depolymerization provided greater total phenolic products, lower selectivities of main products i.e., syringol and acetosyringone were obtained compared with CuFe/Al_2_O_3_ catalyzed the NaOH-lignin depolymerization (Table 2).

For K_2_CO_3_-lignin, the Cu-Fe/SiO_2_ catalyst showed the higher performance and greater selectivity for total phenolic compound production compared with Cu-Fe/Al_2_O_3_ catalyst. Although, Cu-Fe/Al_2_O_3_ catalyst surface contained 8.15 wt% Fe_2_O_3_ and 12.80 wt% CuO similar to 10.38 wt% Fe_2_O_3_ and 12.27 wt% CuO in Cu-Fe/SiO_2_ catalyst (Table 1), nevertheless, the smaller particle size of Cu-Fe/SiO_2_ catalyst analyzed by SEM images (Figure S4) as well as lower acid site density of Cu-Fe/SiO_2_ catalyst compared with that of Cu-Fe/Al_2_O_3_ catalyst (Table S1) substantially promoted the depolymerization of K_2_CO_3_-lignin. From gel permeation chromatography (GPC) results, the K_2_CO_3_-lignin had smaller molecular weight lignin relative to NaOH-lignin [26] and thus particular 2,4-di-tert-butylphenol were selectively generated as the main product (Tables S4 and S5).

In contrast, NaOH-lignin exhibited the greatest amount of syringol and acetosyringone when using Cu-Fe/Al_2_O_3_ as the catalyst from 30-min depolymerization reaction. This was possibly due to the higher molecular weight of NaOH-lignin required stronger acidity of Cu-Fe/Al_2_O_3_ catalyst to facilitate the lignin depolymerization (Table S1). From the results when the oxidative depolymerization took place for 30 min, Cu-Fe/Al_2_O_3_ catalyst exhibited higher selectivity on lignin conversion to both syringol and acetosyringone compared with Cu-Fe/SiO_2_ catalyst. Although, the total phenolic compound from Cu-Fe/Al_2_O_3_ catalyst (49.52 wt%) was lower compared with that from Cu-Fe/SiO_2_ catalyst (63.87 wt%), the higher syringol yield from Cu-Fe/Al_2_O_3_ catalyst (27.07 wt%) was achieved compared with that from Cu-Fe/SiO_2_ catalyst (19.21 wt%). These corresponded to 54.64% and 30.08% selectivity from Cu-Fe/Al_2_O_3_ and Cu-Fe/SiO_2_ catalyst, respectively as demonstrated in Table 2, Tables S6 and S7.

From the main products of lignin depolymerization from NaOH-lignin from EFB i.e., syringol and acetosyringone, similar results were reported for NaOH depolymerized lignin, which contained an increased phenolic hydroxyl group, active protons at C5, and an enhanced methoxyl group twice as much as that of original lignin [45]. In case of K_2_CO_3_-lignin, 65–67% selectivity of 2,4-Di-tert butylphenol was achieved as the main product for the system without catalyst for both 15 min and 30 min of alkaline depolymerization (Table 2). The findings were in good agreement with a previous report in which alkali carbonates influenced a decrease of proton concentration during depolymerization reaction and led to enhancing parallel and secondary reaction mechanisms to generate more phenols and conjugated phenolic compounds from demethylation of original lignin [46].

Table 2 additionally demonstrated the comparison of yield and selectivity of main products from lignin depolymerization, especially syringol and acetosyringone. The findings revealed that homogeneous catalytic depolymerization of EFB lignin by Cu(OH)_2_ + Fe_2_O_3_ gave higher yield and selectivity relative to heterogeneous catalysis. However, similar trends were observed for both homogeneous and heterogeneous depolymerization when highest syringol + acetosyringone yields were achieved when using 15 min of depolymerization for K_2_CO_3_-lignin (50.33 wt% of syringol and 20.48 wt% of acetosyringone) and 30 min depolymerization for NaOH-lignin (52.51 wt% of syringol and 29.58 wt% of acetosyringone). Both conditions provided remarkably high selectivity. Lower selectivity of phenolic compound production indicates that more side reaction products were obtained in the experiments of heterogeneously mixed metal oxides catalysts compared with homogeneous mixed metal oxides catalysts in our previous study [26]. It was observed from GC-MS analysis that when the reaction time was increased from 15 min to 30 min, higher concentration of carboxylic acids and quinone such as benzoic acid and acetic acid were generated Figures S5 and S8.

**Figure 7:**
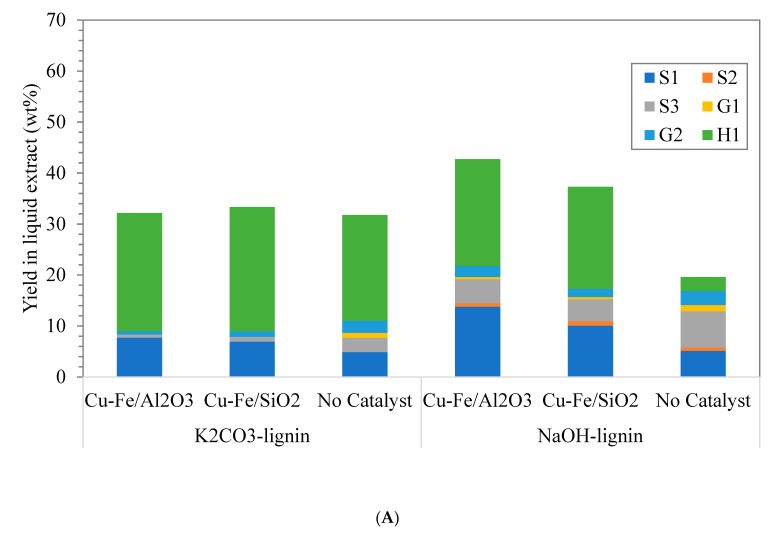

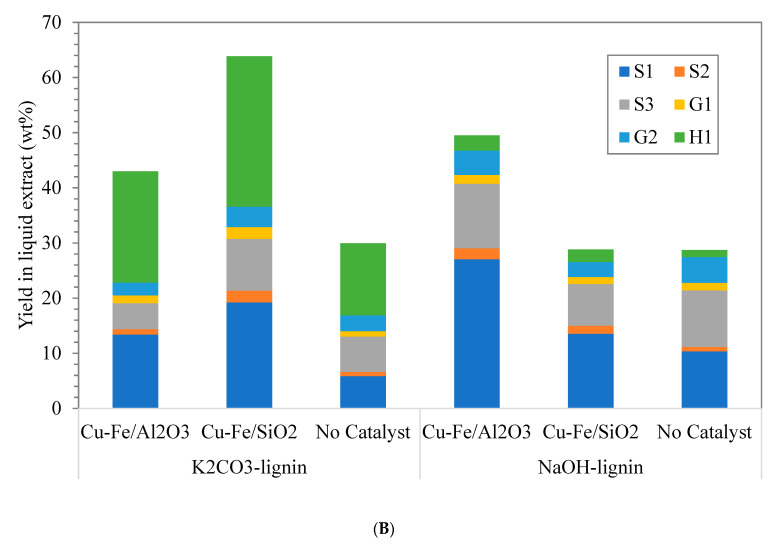
Lignin monomer yield in liquid product from depolymerization of K_2_CO_3_-lignin and NaOH-lignin under microwave heating at 300 W for (**A**) 15 min, and (**B**) 30 min over heterogeneous catalysts namely Cu-Fe/Al_2_O_3_, Cu-Fe/SiO_2_ and without catalyst; S1 = Syringol, S2 = Syringaldehyde, S3 = Acetosyringone, G1 = Vanillin, G2 = Acetovanilone, and H1 = 2,4-Di-tert butylphenol.

As demonstrated in Figure 7, it was obvious that NaOH-lignin from EFB gave higher yield of S-lignin which was mainly syringol and acetosyringone at 15 min of reaction compared with K_2_CO_3_-lignin (Figure 7A), and Cu-Fe/Al_2_O_3_ catalyst markedly facilitated the generation of syringol product over Cu-Fe/SiO_2_ and without catalyst. For the microwave reaction at 30 min, syringol and acetosyringone yields from NaOH-lignin polymerization over Cu-Fe/Al_2_O_3_ and Cu-Fe/SiO_2_ catalysts were substantially enhanced as shown in Figure 7B. This was possibly due to either enhanced hydrogenolysis of β-O-4 ether linkages within lignin precursor or oxidative cleavage of C-O-C under microwave heating over metal catalysts i.e., Fe, Rh which markedly yield S-type lignin relatively to guaiacyl or G-type lignin as main products [13,42]. Another tentative mechanism was oxidative C-O-C break down and demethylation at C_α_ and C_5_ of 2,4-di-tert-butylphenol yielding syringol as a main product.

**Figure 8:**
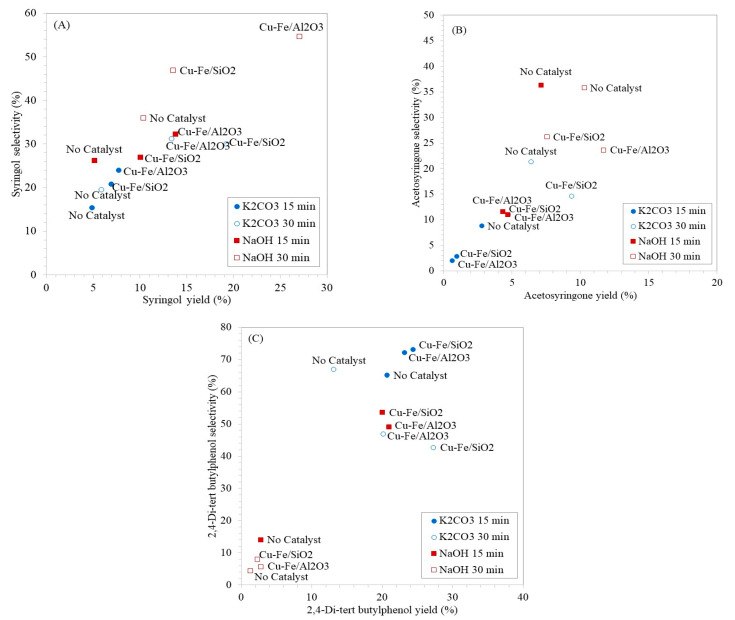
Yield and selectivity from the depolymerization of EFB derived alkaline lignin (K_2_CO_3_-lignin and NaOH-lignin) to (**A**) syringol, (**B**) acetosyringone and (**C**) 2,4-di-tert butylphenol using 300 W microwave reaction for 15 and 30 min over different catalysts.

When considering the yield and selectivity of the main products, Figure 8A–C shows the correlation between the different alkaline extraction methods and the role of heterogeneous catalysts used in the subsequent depolymerization step. In case of syringol production, the depolymerization reaction of NaOH-lignin using Cu-Fe/Al_2_O_3_ catalyst provided the greatest syringol yield (27.06 wt%) and selectivity (54.64 %) from the microwave reaction at 300 W for 30 min as illustrated in Figure 8A. The reason was possibly owing to higher acidity and Fe_2_O_3_ content of Cu-Fe/Al_2_O_3_ catalyst compared with Cu-Fe/SiO_2_ catalyst (Table 1 and Table S1). For production of acetosyringone, NaOH-lignin was the suitable substrate for microwave-assisted depolymerization and the highest monophenolics yield at 10.28 wt% and selectivity at 35.78% were achieved from the reaction at 300 W for 30 min without adding catalyst (Figure 8B). Therefore, mild oxidative reaction using H_2_O_2_ without catalyst was the most optimal condition for acetosyringone production from NaOH-lignin. In case of 2,4-Di-tert butylphenol production (Figure 8C), the highest yield from 23.19–24.39 wt% and selectivity from 72.09–73.11% were obtained from K_2_CO_3_-lignin and successive lignin depolymerization over Cu-Fe/SiO_2_ and Cu-Fe/Al_2_O_3_ catalysts at 300 W for only 15 min. An increase of microwave reaction duration from 15 min to 30 min gave adverse effect on both yield and selectivity of 2,4-Di-tert butylphenol. The results confirmed that the K_2_CO_3_-lignin had smaller molecular weight lignin relative to NaOH-lignin [26] and thus particular 2,4-Di-tert butylphenol was selectively generated as the main products in a very short period of reaction (15 min) over Cu-Fe/SiO_2_ and Cu-Fe/Al_2_O_3_ catalysts.

As shown in Figure 7 and Figure 8, CuFe/Al_2_O_3_ exhibited greater performance on both yield and selectivity toward syringol and acetosyringone, which were the main products of EFB lignin in this system. The synergistic effect of Cu and Fe was found to favor the reactivity of the catalyst. The results were confirmed by greater monophenolic yield and selectivity of the products. The present system gave superior phenolic yields compared with other previous work on lignin depolymerization, for example 17.92 wt% monophenolic compound from CuO/Fe_2_(SO_4_)_3_/NaOH catalyst [61], less than 35 wt% monophenolic yield from CuSO_4_ and LaMn_0.__8_Cu_0.__2_O_3_ catalysts [34].

**Figure 9:**
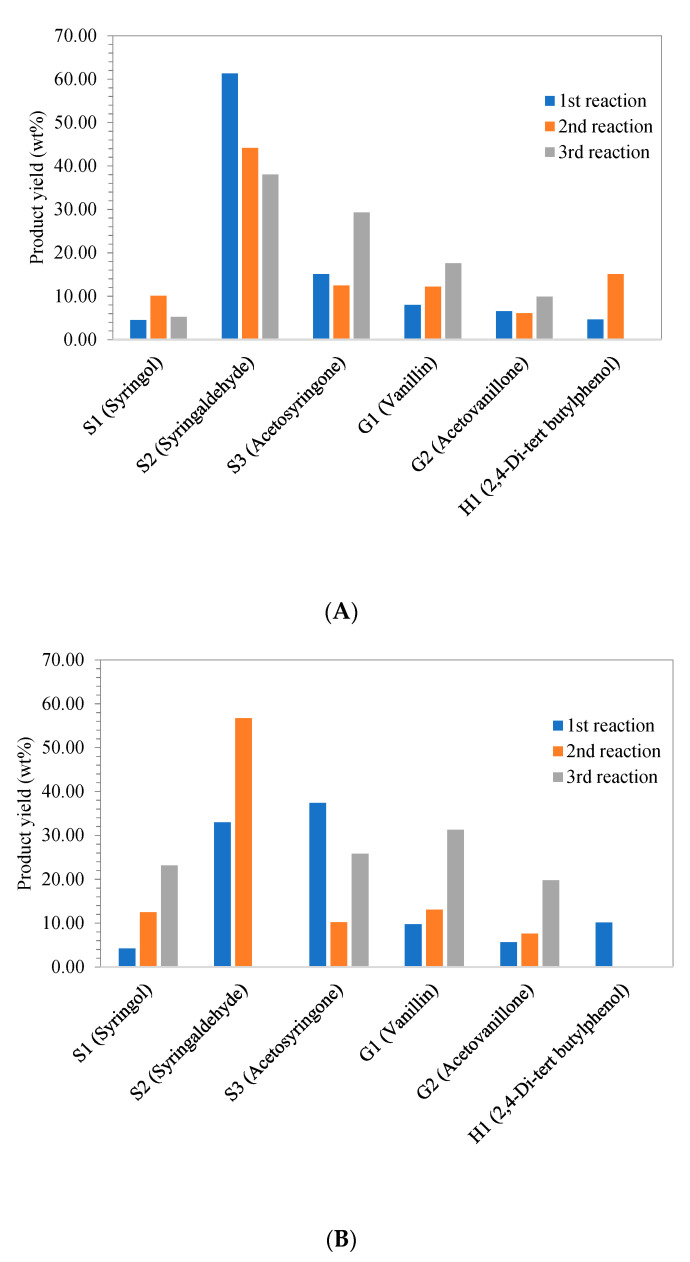
Product yield from the recyclability study of (**A**) CuFe/Al_2_O_3,_ and (**B**) CuFe/SiO_2_ catalysts on depolymerization of NaOH-lignin under microwave at 300 W for 30 min.

**Figure 10:**
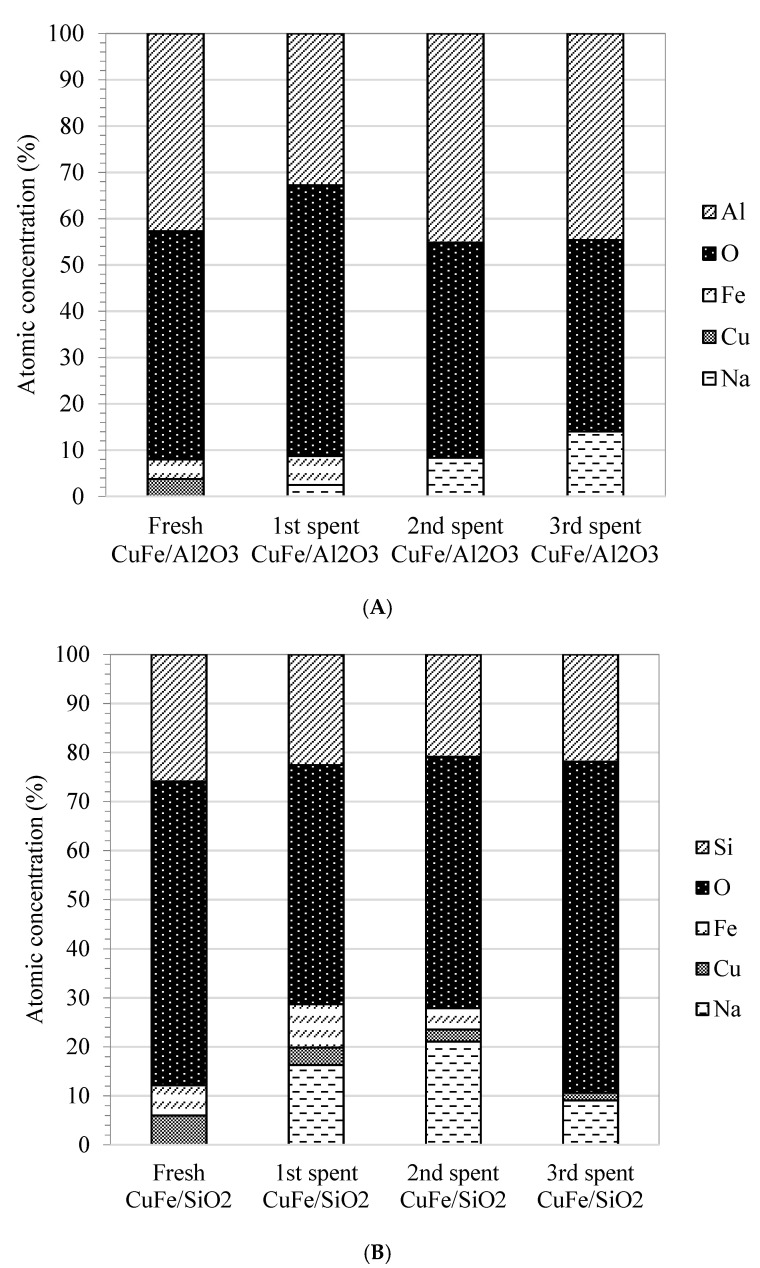
Atomic concentration of fresh and spent catalysts (**A**) CuFe/Al_2_O_3_ and (**B**) CuFe/SiO_2_ from recyclability study of heterogeneous catalyst on NaOH-lignin depolymerization under microwave heating at 300 W for 30 min.

From recyclability study, the amount of main products from fresh and spent catalysts was quantified using standard curve (Figure S9). The results from Figure 9A showed that the presence of Fe and Cu on Al_2_O_3_ support from CuFe/Al_2_O_3_ catalyst favored to produce high yield of syringaldehyde from NaOH-lignin in the 1st reaction in which fresh catalyst was used. However, the 2nd and 3rd reaction of spent catalyst gave minimal yield of syringaldehyde in a respective degree (Table S8) due to the leaching of Cu and Fe respectively as demonstrated in XPS analysis results for Fe2p and Cu2p of spent CuFe/Al_2_O_3_ catalyst in Figure 10A. After Cu and Fe leaching, acidity of Al_2_O_3_ support seemingly enhanced the yield of acetosyringone, vanillin, and acetovanillone. Similar to CuFe/SiO_2_ catalyst, fresh catalyst was prone to selectively generate acetosyringone and syringaldehyde as demonstrated in Figure 9B. The spent CuFe/SiO_2_ catalyst was found to lose Cu and Fe respectively during the second time of recyclability test (Figure 10B), therefore the effect of SiO_2_ support was found to favor vanillin, acetosyringone, syringol, and acetovanillone as NaOH-lignin depolymerization products in a respective degree. SiO_2_ support exhibited no effect on generation of syringaldehyde and (2,4-Di-tert butylphenol) without Cu and Fe doping.

### 3.4. The Proposed Mechanism of Oxidative Depolymerization of EFB Derived Lignin with Mixed Metal Oxides Cu-Fe Catalyst

The results of the present experiments were consistent with a previous report of Ma and coworkers [32] who reported that catalysts of Cu (II), Fe (III), and Mn (II, III) played an important role in catalysis of oxidation reaction of lignin structure in the presence of oxygen or peroxide (H_2_O_2_). By breaking down the β-O-4 bonds in the lignin structure via oxidative and hydrolysis reaction, the lignin structure was depolymerized to monophenolic compounds such as vanillin, syringaldehyde or *p*-hydrobenzaldehyde. Similarly, Ouyang studied the Cu(II) and Fe(III) catalyzed reactions in alkaline solution for lignin depolymerization that were able to produce a high yield of phenolic compounds [61]. It was postulated that the oxidation of lignin structure does not only cleave the β-O-4 or C-C bonds in lignin, but also breaks down the structure of the aromatic ring resulting in smaller phenolic monomers such as phenol and benzoic acid. It additionally produced by-products including quinones and dicarboxylic acid groups such as formic acid, acetic acid and butanoic acid by ring-opening reactions (Figures S3–S6).

EFB lignin contains a substantial fraction of sinapyl units, which can be observed from syringol derivatives after oxidative depolymerization. From the results, syringaldehyde, acetosyringone, acetovanillone, and vanillin were the major products formed during lignin depolymerization. The lignin oxidative degradation results indicate that the transformation mechanism of lignin could generate oligomers, and subsequent phenolic compounds involving a free radical pathway that initiates cleavage of alkyl-aryl ether (α-O-4 and β-O-4), aryl-aryl ether (4-O-5) and aryl-aryl (5-5) bonds, hydrogen abstraction and β-scission reactions, which is in good agreement with previous work [62]. It was found that similar products were detected from lignin depolymerization via pyrolysis and UV radiation. It can be implied that thermal energy is the main driving force for the aforementioned bond fission reactions in thermolysis, while UV radiation augments the bond cleavage in photocatalysis. In the present study, microwave radiation and the reactive radical species such as •OH and O_2_•^−^ radicals from H_2_O_2_ dissociation induce these reactions to occur. Importantly, hydroxyl radicals can react with benzene ring via electrophilic addition and cause the cleavage of α-O-4 or β-O-4 ether links in lignin [63]. As a result, OH group substitution is achieved. Moreover, the previous research reported that the formation of dimethoxy benzoquinone was earlier proposed to occur by the action of singlet oxygen (^1^O_2_) or superoxide radicals (O_2_•^–^) on the phenolic ring, which results in the cleavage of the bond between aromatic and the α-carbon [63]. Solely the effect of either Cu or Fe did not influence the improvement of the reaction, but the combination effect of bimetallic Cu-Fe catalyst. This was confirmed by the findings from a previous work demonstrating that Fe_2_O_3_/γ-Al_2_O_3_ catalyst provided similar lignin degradation product and yield similar with the blank test. The Fe_2_O_3_/γ-Al_2_O_3_ catalyst did not show good activity in the lignin oxidation reaction [64].

The aforementioned phenomena were found to give superior catalytic performance from the synergistic effect of bimetallic Cu and Fe, especially on Al_2_O_3_ support. It has been observed that the oxygen space will be enhanced with the partial replacement of Fe^3+^ by Cu^2+^, according to the previous report [65], which would accelerate the oxygen surface absorption ability of the catalyst and the intermediate content of O_2_-Fe^3+^-lignin complex will be enhanced [66]. They act as oxygen carriers that can attack the lignin [67]. Moreover, the amount of activated species Cu^2^ + O_2_^−^ will be increased with the partial replacement of Fe^3+^ by Cu^2+^, which will result in a cycling of Cu^2+^/Cu^+^ (Cu^2+^→Cu^+^→Cu^2^ + O_2_^−^→Cu^2+^) and Fe^3+^/Fe^2+^ [23]. The proposed mechanism was in good accordance with XPS (Fe2p and Cu2p) and XRF results, which indicated the presence of CuO/Cu_2_O and Fe_2_O_3_/FeO, respectively. This cycling accelerates the generation of the intermediate quinone methide radicals [68]. Moreover, the intermediate reduction potential of Cu^2+^ found in alkaline condition (−0.16 V for the CuO/Cu_2_O redox pair at pH 14) was postulated to be satisfactory for oxidation of lignin to aldehydes with limited subsequent oxidation of aldehydes [27]. With all the combined effect of the above mentioned factors, the catalytic activity of CuFe/Al_2_O_3_ is improved.

The role of catalyst support was proved in the recyclability study. The previous study revealed that relatively more acidic γ-Al_2_O_3_ support showed better catalyst performance than CeO_2_ or TiO_2_ to generate vanillin from lignin depolymerization [30,64]. As a result, in the present study, SiO_2_ had higher acidity than Al_2_O_3_, and therefore played a vital role to enhance the conversion of guaiacyl lignin (G-lignin) to form acetovanillone and vanillin relatively to Al_2_O_3_ as demonstrated in Figure 9 for the 3rd reaction when Cu and Fe were leached out. In the case of SiO_2_ support, it was additionally postulated that H_2_O_2_ decomposition formed reactive oxygen species and are then physisorbed on silica framework trapped on the hydroxyl network, and eventually transferred to the secondary carbon on the side chain. Consequently, oxidation to such secondary carbon converts it to a more stable carbonyl group of acetovanillone. Further oxidation could yield vanillin as the final product. As shown in Figure 9B, the 3rd spent CuFe/SiO_2_ catalyst with the leaching of Cu and Fe indicated by decreased intensity of XPS (Cu2p and Fe2p) could significantly change the reaction pathway to more selectively generate acetovanillone and vanillin. The reason was confirmed by a previous study on lignin model compound depolymerization using various structure of silica catalyst under microwave irradiation [69] revealing that surface hydroxyl groups, which in turn facilitate the adsorption of 4-hydroxy-3-methoxy-alpha-methyl benzylalcohol or apocynol leading to high conversion to acetovanillone in the systems. Similar result was observed in the case of Al_2_O_3_ support. After Cu and Fe leaching, effect of acidity of solely Al_2_O_3_ seemingly shifted the selectivity of product from syringaldehyde to acetovanillone and vanillin as demonstrated in Figure 9A and Figure 10A. 

From the lignin depolymerization with mixed metal oxides catalyst, the 2,4-di-tert-butylphenol was one of the different major products produced in the reaction mixture. This has been shown to occur during lignin degradation by mixed metal oxide catalysts typically containing aluminum (Al_2_O_3_) and silicon (SiO_2_) as active sites for promoting chemical reactions [70]. However, their reactivity to breakdown inter-unit linkages remains to be proven. It has been revealed that under mild oxidative lignin depolymerization, the side-chain hydroxyl groups were oxidized to carbonyl groups, and after that the reaction is quenched. This conceivably provides a highly selective lignin oxidative modification and warrants further investigation [32,70]. Based on the previous study, mixed Cu-Fe oxide catalyst can possibly react with the electronegative hydroxyl groups of H_2_O_2_ and H_2_O, and thus remove the hydroxyl group from lignin monomer. The partial hydrogenation of the benzene ring intermediates is postulated, which is favorable to the subsequent dehydroxylation due to the lower bond dissociation energy [71,72]. The intermediate product then reacts with the adsorbed methyl groups, leading to the formation of primitive alkylphenol. The methyl group can be formed from the demethylation step during guaiacol generated during lignin depolymerization [73]. Subsequently, the higher alkylphenols, including tert-butylphenols, iso-propylphenols, and neo-pentylphenols could be formed [74].

## 4. Conclusions

Lignin depolymerization was successfully catalyzed by Cu (II) and Fe (III) mixed metal oxides catalyst supported on Al_2_O_3_ and SiO_2_ support. The highest percentage of total phenolic compounds of 63.87 wt% was obtained from microwave-induced oxidative degradation of K_2_CO_3_-lignin when the lignin depolymerization reaction carried out at 300 W, 30 min with 1.0 wt% H_2_O_2_ and catalyzed by Cu-Fe/SiO_2_ catalyst. However, when the main products were considered, it contained 19.21 wt% of syringol corresponding to 30.08% selectivity. In contrast, the Cu-Fe/Al_2_O_3_ catalyst gave lower total phenolic compounds of 49.52 wt% from NaOH-lignin, but it provided the greatest selectivity of syringol and acetosyrigone at 54.64% and 23.65%, respectively (78.29% total selectivity of two products). Consequently, this optimal condition successfully generated the most favorable value-added chemicals from EFB lignin for utilization as food aroma additives and chemical feedstock.

## Figures and Tables

**Table 1:** The percentage of metal oxides in heterogeneously Cu-Fe/Al_2_O_3,_ Cu-Fe/SiO_2_ mixed metal oxides catalysts and SiO_2_, Al_2_O_3_ supports analyzed by X-ray Fluorescence Spectrometry (XRF).

Element (wt%)	Catalyst			
	Cu-Fe/Al_2_O_3_	Cu-Fe/SiO_2_	Al_2_O_3_	SiO_2_
CuO	12.80	12.27	nd	nd
Fe_2_O_3_	8.15	10.38	0.02	0.05
Al_2_O_3_	78.67	0.12	99.57	0.15
SiO_2_	0.07	76.36	0.12	98.54
Others	0.31	0.87	0.29	1.26

nd = not detected.

**Table 2:** Percentage of phenolic compounds concentration from GC-MS analysis for K_2_CO_3_-lignin and NaOH-lignin depolymerization with 1.0 wt% H_2_O_2_ at 300 W using Cu-Fe/Al_2_O_3_, Cu-Fe/SiO_2_, and without catalyst.

Type of Reaction	Alkaline EFB Extracted Lignin	Catalyst	Main Products (wt%)						Total Phenolic Compounds (wt%)	%Selectivity			Ref.
			Syringol 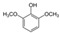	Vanillin 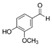	Aceto-Vanillone 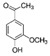	2,4-Di-tert Butylphenol 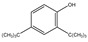	Syringal-Dehyde 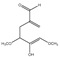	Aceto Syringone 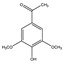		Syringol 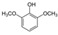	2,4-Di-tert Butylphenol 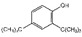	Acetosyringone 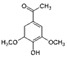	
Heterogeneous reaction ^a^	15 min												
	K_2_CO_3_-lignin	Cu-Fe/Al_2_O_3_	7.71	-	0.63	23.19	-	0.64	32.17	23.97	72.09	1.99	This study
		Cu-Fe/SiO_2_	6.94	-	1.08	24.39	-	0.95	33.36	20.80	73.11	2.85	This study
		No Catalyst	4.89	0.97	2.40	20.71	-	2.80	31.77	15.39	65.19	8.81	This study
	NaOH-lignin	Cu-Fe/Al_2_O_3_	13.78	0.45	2.13	20.98	0.68	4.71	42.73	32.25	49.10	11.02	This study
		Cu-Fe/SiO_2_	10.07	0.41	1.60	20.02	0.87	4.33	37.30	27.00	53.67	11.61	This study
		No Catalyst	5.14	1.19	2.76	2.76	0.65	7.12	19.62	26.20	14.07	36.29	This study
	30 min												
	K_2_CO_3_-lignin	Cu-Fe/Al_2_O_3_	13.39	1.39	2.33	20.17	0.98	4.72	42.98	31.15	46.93	10.98	This study
		Cu-Fe/SiO_2_	19.21	2.16	3.69	27.29	2.16	9.36	63.87	30.08	42.73	14.65	This study
		No Catalyst	5.86	1.00	2.84	13.09	0.77	6.39	29.95	19.57	66.89	21.34	This study
	NaOH-lignin	Cu-Fe/Al_2_O_3_	27.06	1.61	4.39	2.78	1.97	11.71	49.52	54.64	5.61	23.65	This study
		Cu-Fe/SiO_2_	13.52	1.25	2.74	2.29	1.48	7.56	28.84	46.88	7.94	26.21	This study
		No Catalyst	10.34	1.35	4.7	1.27	0.79	10.28	28.73	35.99	4.42	35.78	This study
Homogeneous reaction ^b^	15 min												
	K_2_CO_3_-lignin	Cu(OH)_2_ + Fe_2_O_3_	50.33	3.24	10.72	-	4.96	20.48	89.73	56.09	-	22.82	[26]
	NaOH-lignin	Cu(OH)_2_ + Fe_2_O_3_	28.11	1.39	4.22	-	3.36	7.55	44.63	62.98	-	16.92	[26]
	30 min												
	K_2_CO_3_-lignin	Cu(OH)_2_ + Fe_2_O_3_	44.77	4.00	10.15	-	6.66	22.52	88.1	50.82	-	25.56	[26]
	NaOH-lignin	Cu(OH)_2_ + Fe_2_O_3_	52.51	3.89	8.23	-	4.84	19.58	89.05	58.97	-	21.99	[26]

^a^ The reaction was carried out under microwave reactor at 300 W for 15 or 30 min for 0.3 g K_2_CO_3_-lignin and NaOH-lignin, 0.15 g of heterogeneously mixed metal oxide catalyst and 1 wt% of H_2_O_2_ in 3 mol L^−1^ NaOH solution. ^b^ Lignin (0.3 g) was added into a microwave reactor containing a H_2_O_2_ (1 wt%, 2 mL) and NaOH (3 mol L^−1^, 14 g) solution with the presence of catalysts (0.02 g of Cu(OH)_2_ and 0.002 g of Fe_2_O_3_). The reaction took place at 300 W for 15 min or 30 min.

## Supplementary Materials

The following are available online. Table S1: Acidity of synthesized catalysts from NH_3_-TPD analysis, Table S2: The type of element from EDX analysis of Cu-Fe/Al_2_O_3_ catalyst, Table S3: The type of element from EDX analysis of Cu-Fe/SiO_2_ catalyst, Table S4: The phenolic compounds peak area percentage from GC-MS analysis for K_2_CO_3_-lignin depolymerization with Cu-Fe/Al_2_O_3_, Cu-Fe/SiO_2_, and without catalyst (microwave heating at 300 watts, 1% *w*/*w* of H_2_O_2_ in NaOH solution for 15 min), Table S5: The phenolic compounds peak area percentage from GC-MS analysis for K_2_CO_3_-lignin depolymerization with Cu-Fe/Al_2_O_3_, Cu-Fe/SiO_2_, and without catalyst (microwave heating at 300 watts, 1% *w*/*w* of H_2_O_2_ in NaOH solution for 30 min), Table S6: The phenolic compounds concentration peak area from GC-MS analysis for NaOH-lignin depolymerization with Cu-Fe/Al_2_O_3_, Cu-Fe/SiO_2_, and without catalyst (microwave heating at 300 watts, 1% w/w of H_2_O_2_ in NaOH solution for 15 min), Table S7: The phenolic compounds concentration peak area from GC-MS analysis for NaOH-lignin depolymerization with Cu-Fe/Al_2_O_3_, Cu-Fe/SiO_2_ and without catalyst (microwave heating at 300 watts, 1% w/w of H_2_O_2_ in NaOH solution for 30 min); Table S8: Recyclability study of CuFe/Al_2_O_3_ and CuFe/SiO_2_ catalysts on depolymerization of NaOH-lignin under microwave at 300 W for 30 min; Figure S1: NH_3_-TPD chromatograms of synthesized catalysts and supports of (a) Cu-Fe/Al_2_O_3_, (b) Al_2_O_3_, (c) Cu-Fe/SiO_2_, (d) SiO_2_, Figure S2: The elemental composition of Cu-Fe/Al_2_O_3_ mixed metal oxide catalyst from EDX analysis, Figure S3: The elemental composition of Cu-Fe/SiO_2_ mixed metal oxide catalyst from EDX analysis, Figure S4: Morphological of heterogeneous bimetallic and metal organic framework catalysts (a) Cu-Fe/Al_2_O_3_ at ×500 magnification (b) Cu-Fe/Al_2_O_3_ ×1000 magnification (c) Cu-Fe/SiO_2_ at ×500 magnification, and (d) Cu-Fe/SiO_2_ at ×2000 magnification, Figure S5: The phenolic compounds concentration peak area from GC-MS analysis for K_2_CO_3_-lignin depolymerization with Cu-Fe/Al_2_O_3_, Cu-Fe/SiO_2_, and without catalyst (Microwave heating at 300 watts, 1% *w*/*w* of H_2_O_2_ in NaOH solution for 15 min), Figure S6: The phenolic compounds concentration peak area from GC-MS analysis for K_2_CO_3_-lignin depolymerization with Cu-Fe/Al_2_O_3_, Cu-Fe/SiO_2_, and without catalyst (microwave heating at 300 watts, 1% *w*/*w* of H_2_O_2_ in NaOH solution for 30 min), Figure S7: The phenolic compounds concentration peak area from GC-MS analysis for NaOH-lignin depolymerization with Cu-Fe/Al_2_O_3_, Cu-Fe/SiO_2_, and without catalyst (microwave heating at 300 watts, 1% *w*/*w* of H_2_O_2_ in NaOH solution for 15 min), Figure S8: The phenolic compounds concentration peak area from GC-MS analysis for NaOH-lignin depolymerization with Cu-Fe/Al_2_O_3_, Cu-Fe/SiO_2_, and without catalyst (microwave heating at 300 watts, 1% *w*/*w* of H_2_O_2_ in NaOH solution for 30 min), Figure S9: Standard curve from GC analysis of main products of lignin depolymerization for recyclability study.
