# Supplementary material for: Lignin-Derived Syringol and Acetosyringone from Palm Bunch Using Heterogeneous Oxidative Depolymerization over Mixed Metal Oxide Catalysts under Microwave Heating
Source: Molecules. 2021 Dec 8;26(24):7444. doi: 10.3390/molecules26247444 (PMC8707958; doi:10.3390/molecules26247444)
Supplement: Supplementary file 1 [file molecules-26-07444-s001.zip › molecules-1409819-supplementary.pdf]

Supporting Information

Lignin-Derived Syringol and Acetosyringone from Palm Bunch Using Heterogeneous Oxidative Depolymerization over Mixed Metal Oxide Catalysts Under Microwave Heating

Rangsalid Panyadee [1], Aphinan Saengsrichan [1], Pattaraporn Posoknistakul [1], Navadol Laosiripojana [2], Sakhon Ratchahat [1], Babasaheb M. Matsagar [3], Kevin C.-W. Wu [3,4,5] and Chularat Sakdaronnarong [1,*]

[1] Department of Chemical Engineering, Faculty of Engineering, Mahidol University, 999 Putthamonthon 4 Road, Salaya, Putthamonthon, Nakorn Pathom 73170, Thailand; rangsalid.p@gmail.com (R.P.); aphinan.sae@student.mahidol.edu (A.S.); pattaraporn.pos@mahidol.edu (P.P.); sakhon.rat@mahidol.edu (S.R.); chularat.sak@mahidol.ac.th (C.S.)

[2] The Joint Graduate School of Energy and Environment (JGSEE), King Mongkut’s University of Technology Thonburi, 126 Pracha Uthit Road, Bang Mot, Tungkru, Bangkok 10140, Thailand; navadol@jgsee.kmutt.ac.th

[3] Department of Chemical Engineering, National Taiwan University, No.1, Sec.4 Roosevelt Road,
Taipei City 10617, Taiwan; bmatsagar22@gmail.com (B.M.M.); kevinwu@ntu.edu.tw (K.C.-W.W.)

[4] Center of Atomic Initiative for New Materials (AI-MAT), National Taiwan University,
Taipei City 10617, Taiwan

[5] International Graduate Program of Molecular Science and Technology, National Taiwan University (NTU), Taipei City 10617, Taiwan

***** Correspondence: chularat.sak@mahidol.ac.th

Table S1 Acidity of synthesized catalysts from NH_3-TPD analysis.

**Catalysts**	**Total acid site density (mmol g^{-1})**
Cu-Fe/Al_2O_3	0.384
Al_2O_3	0.534
Cu-Fe/SiO_2	0.251
SiO_2	0.967

(a) Cu-Fe/Al_2O_3

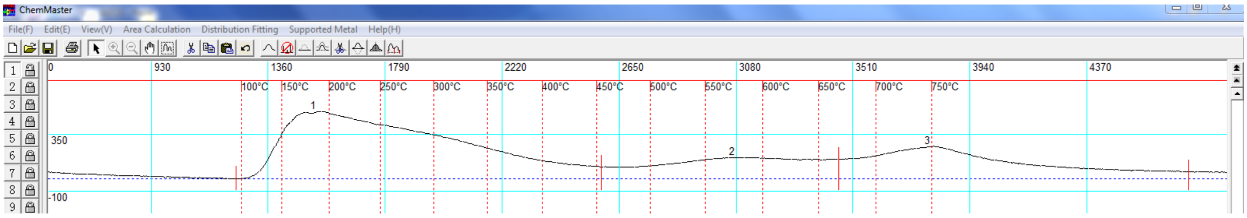

(b) Al_2O_3

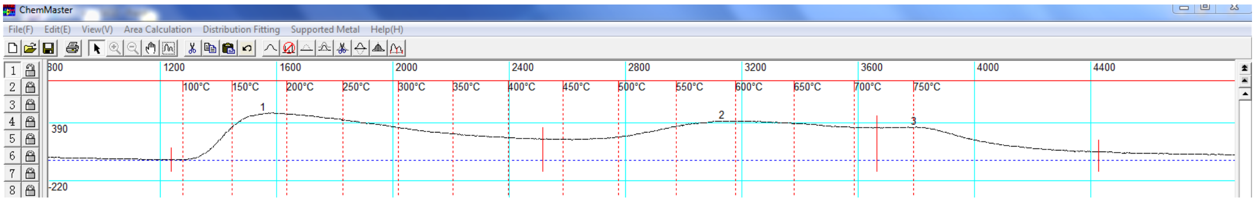

(c) Cu-Fe/SiO_2

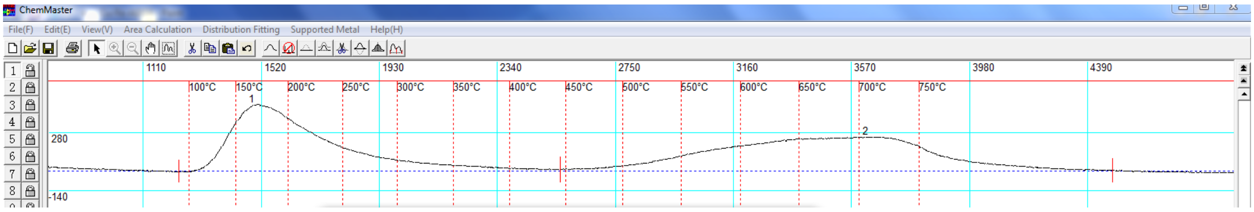

(d) SiO_2

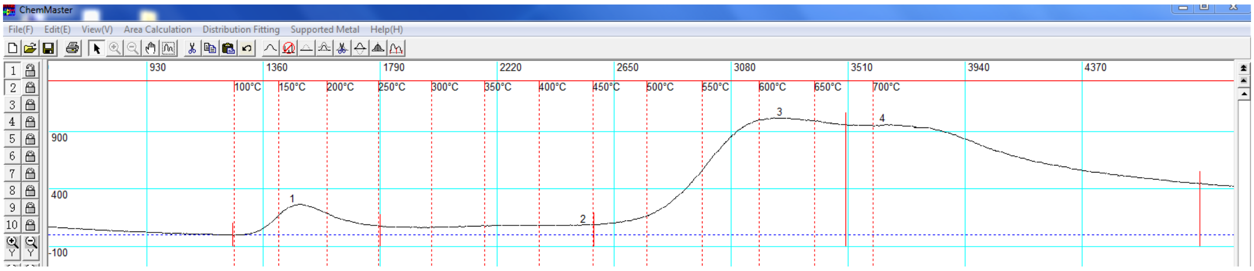

Figure S1 NH_3-TPD chromatograms of synthesized catalysts and supports of (a) Cu-Fe/Al_2O_3, (b) Al_2O_3, (c) Cu-Fe/SiO_2, (d) SiO_2.

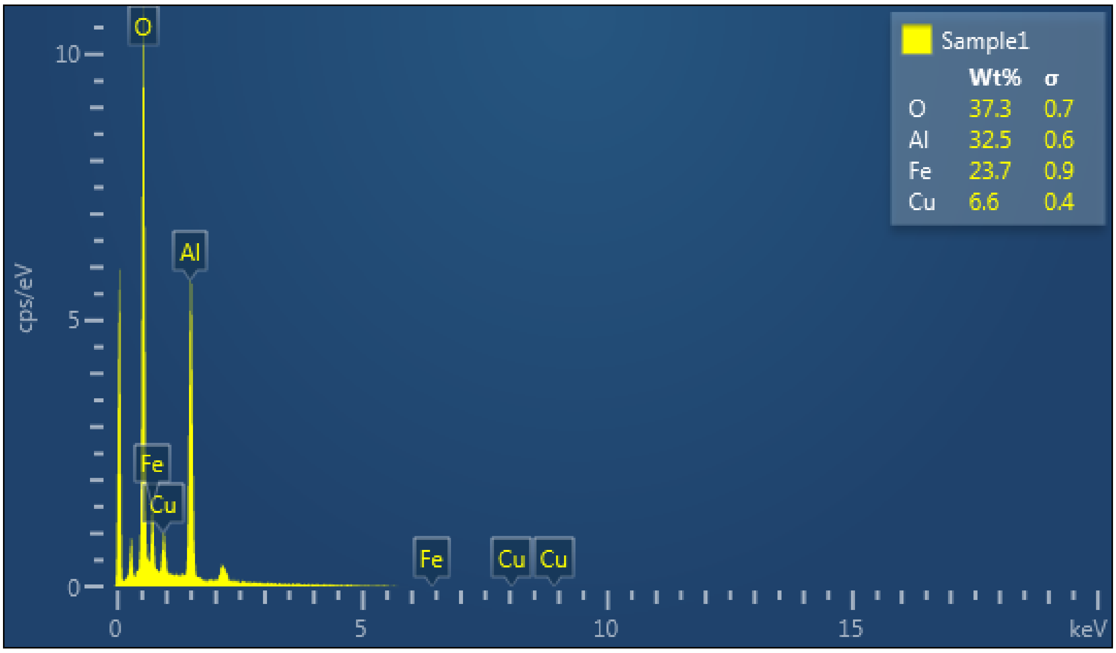

Figure S2 The elemental composition of Cu-Fe/Al_2O_3 mixed metal oxide catalyst from EDX analysis.

Table S2 The type of element from EDX analysis of Cu-Fe/Al_2O_3 catalyst

Element	Line Type	Apparent Concentration	k Ratio	wt%	wt% Sigma	Oxide	Oxide %	Standard Label
O	-	-	-	37.31	-	-	-	-
Al	K series	14.84	0.10656	32.46	0.57	Al_2O_3	61.34	Al_2O_3
Fe	L series	6.17	0.06166	23.66	0.92	FeO	30.44	Fe
Cu	L series	1.56	0.01563	6.57	0.37	CuO	8.22	Cu
Total:	-	-	-	100.00	-	-	100.00	-

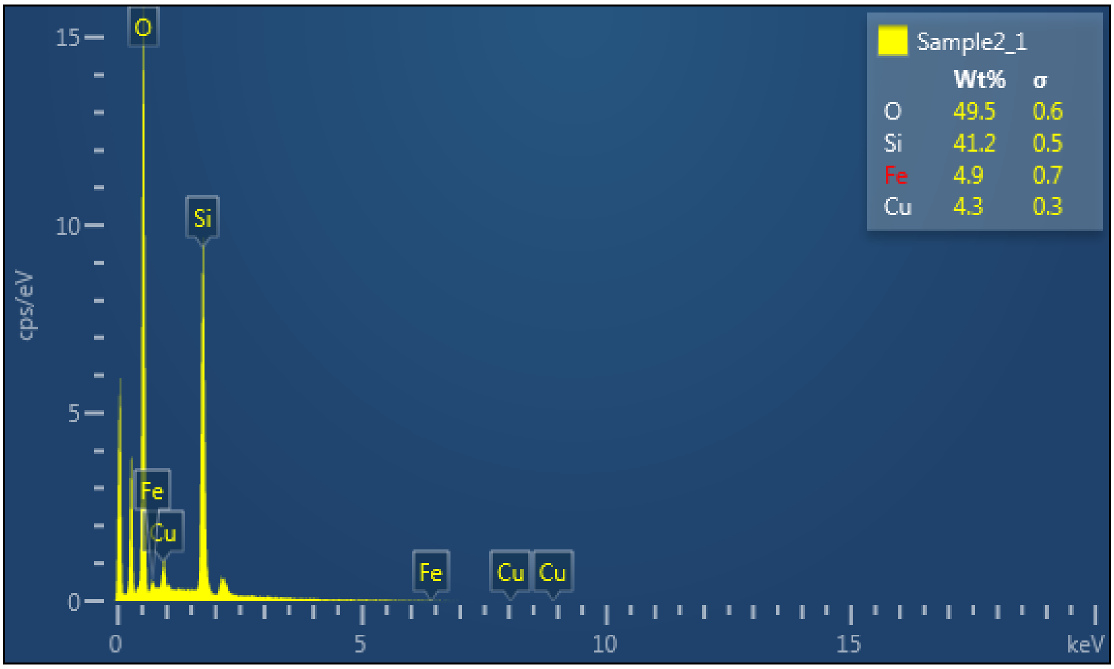

Figure S3 The elemental composition of Cu-Fe/SiO_2 mixed metal oxide catalyst from EDX analysis.

Table S3 The type of element from EDX analysis of Cu-Fe/SiO_2 catalyst

Element	Line Type	Apparent Concentration	k Ratio	Wt%	Wt% Sigma	Oxide	Oxide %	Standard Label
O				49.49				
Si	K series	20.90	0.16557	41.25	0.50	SiO_2	88.24	SiO_2
Fe	L series	1.28	0.01281	4.91	0.71	FeO	6.31	Fe
Cu	L series	1.23	0.01232	4.35	0.28	CuO	5.44	Cu
Total:				100.00			100.00	

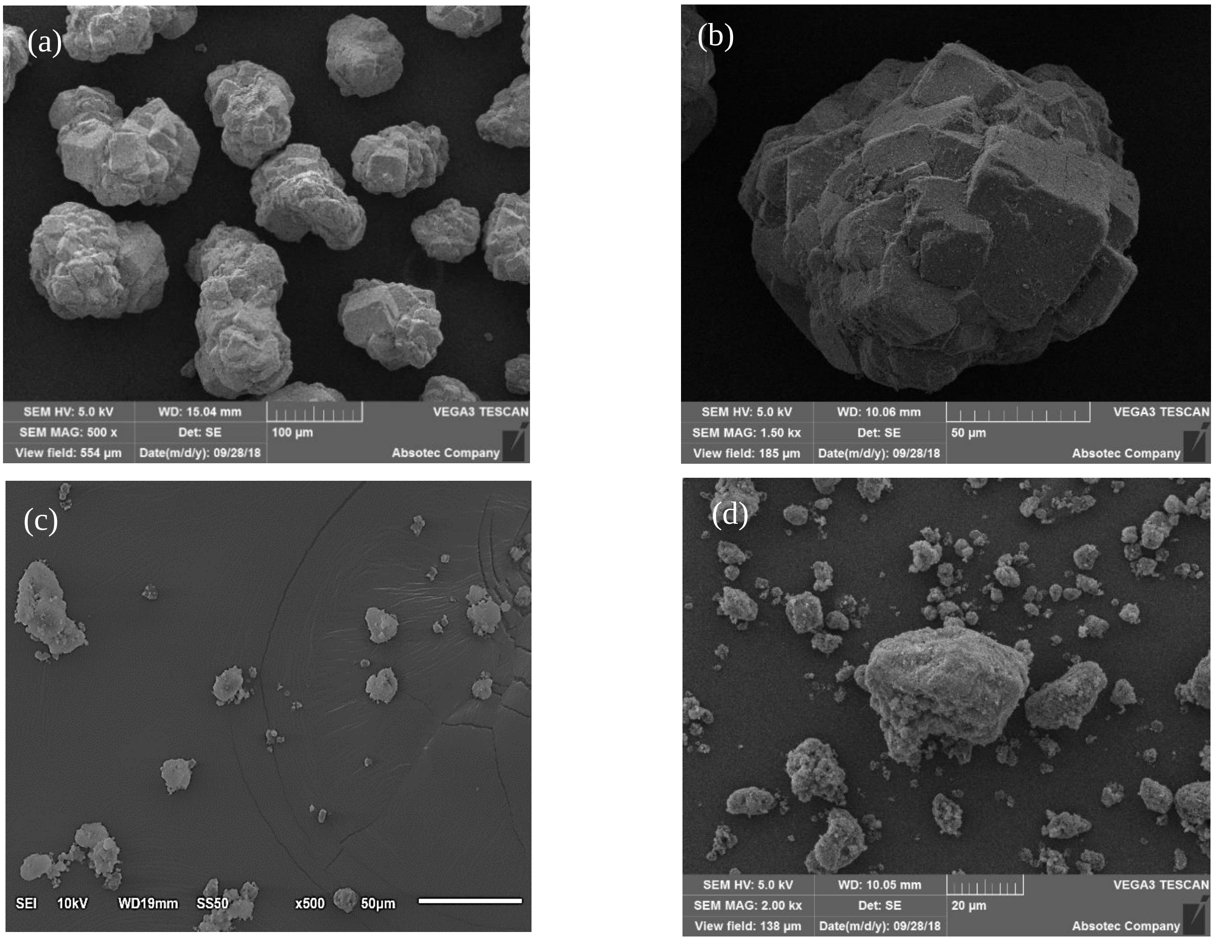

Figure S4 Morphological of heterogeneous bimetallic and metal organic framework catalysts (a) Cu-Fe/Al_2O_3 at ×500 magnification (b) Cu-Fe/Al_2O_3 ×1000 magnification (c) Cu-Fe/SiO_2 at ×500 magnification, and (d) Cu-Fe/SiO_2 at ×2000 magnification.

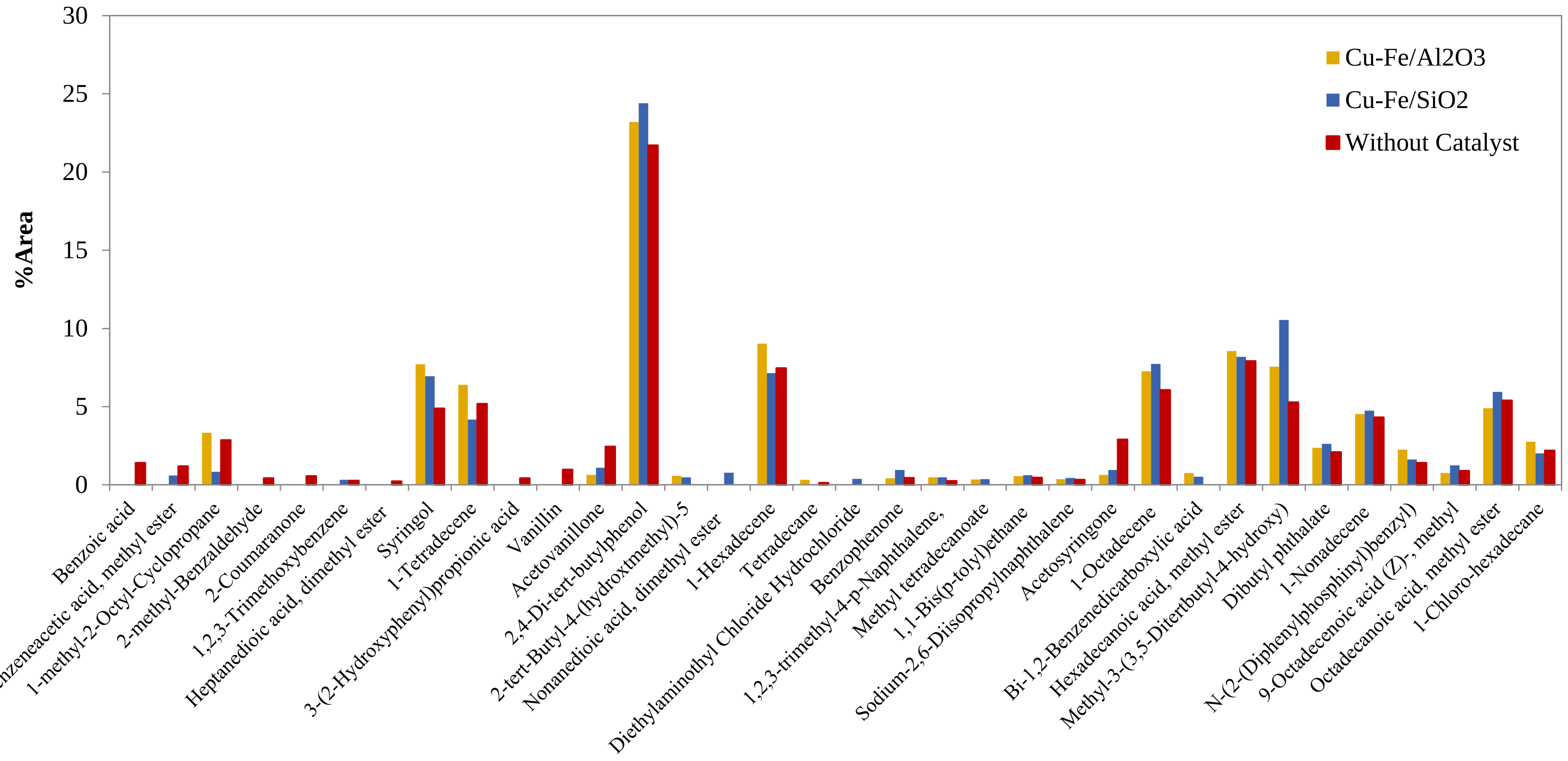

Figure S5 The phenolic compounds concentration peak area from GC-MS analysis for K_2CO_3-lignin depolymerization with Cu-Fe/Al_2O_3, Cu-Fe/SiO_2, and without catalyst (Microwave heating at 300 watts, 1%w/w of H_2O_2 in NaOH solution for 15 min)

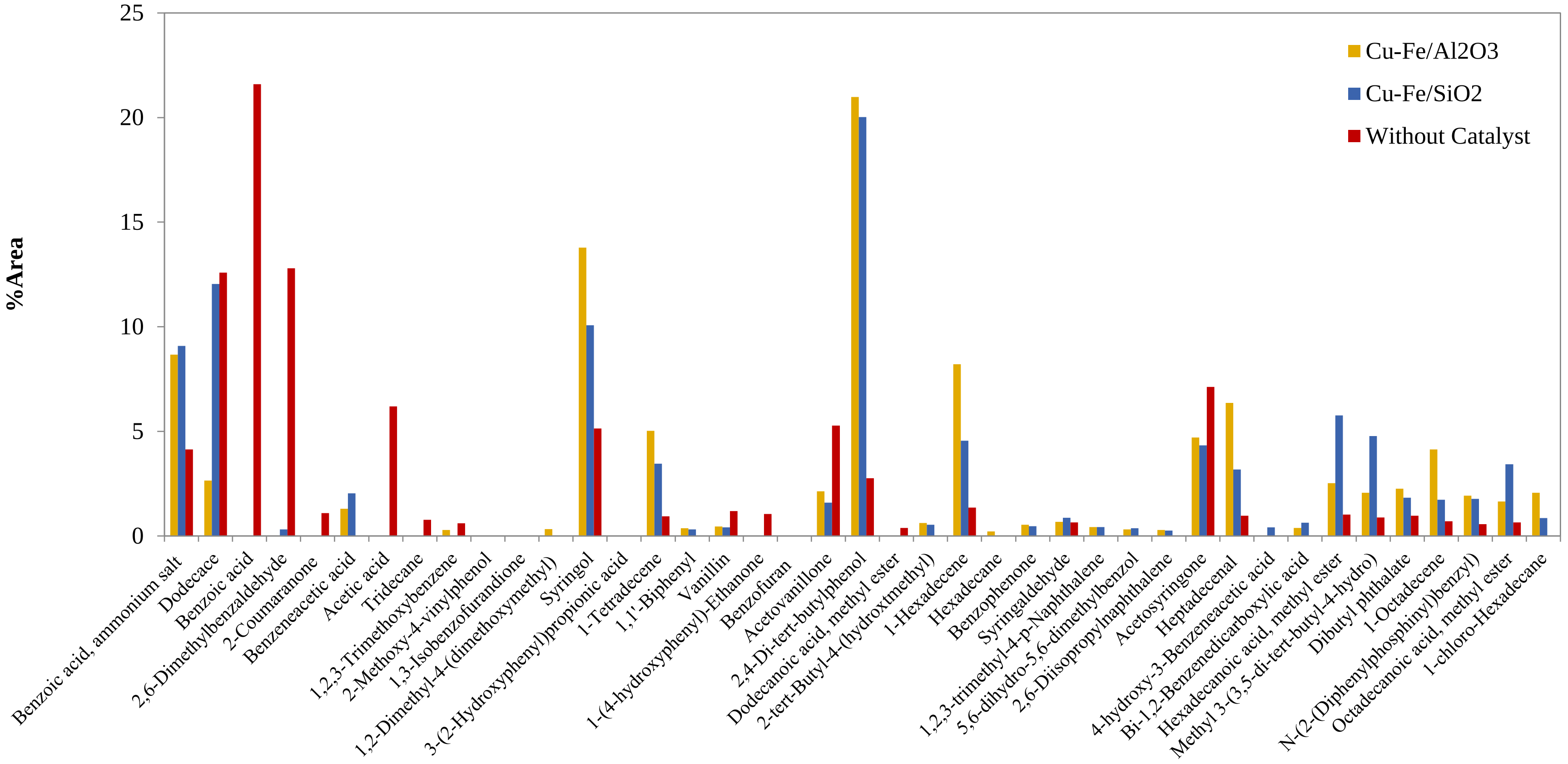

Figure S6 The phenolic compounds concentration peak area from GC-MS analysis for K_2CO_3-lignin depolymerization with Cu-Fe/Al_2O_3, Cu-Fe/SiO_2, and without catalyst (microwave heating at 300 watts, 1%w/w of H_2O_2 in NaOH solution for 30 min).

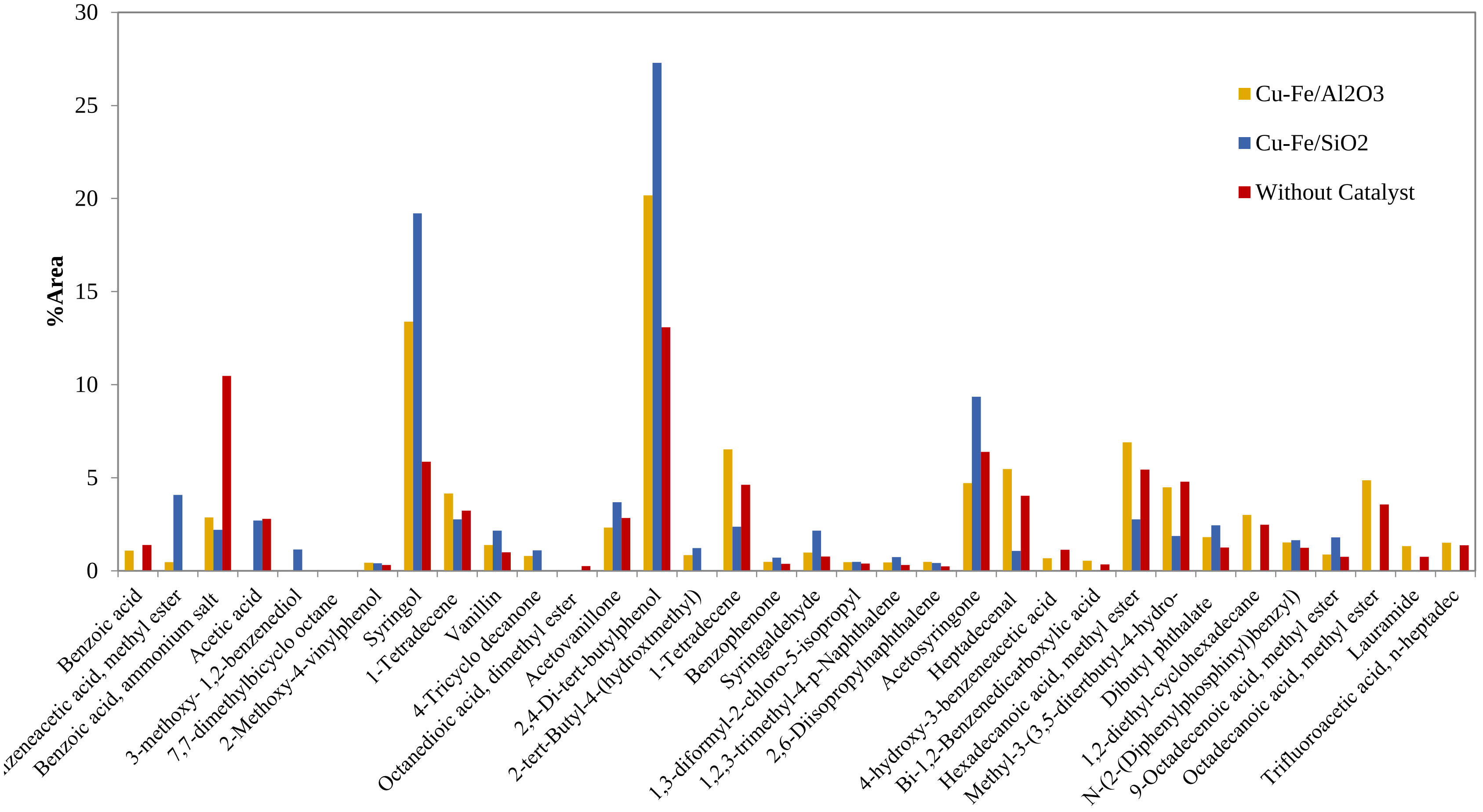

Figure S7 The phenolic compounds concentration peak area from GC-MS analysis for NaOH-lignin depolymerization with Cu-Fe/Al_2O_3, Cu-Fe/SiO_2, and without catalyst (microwave heating at 300 watts, 1%w/w of H_2O_2 in NaOH solution for 15 min).

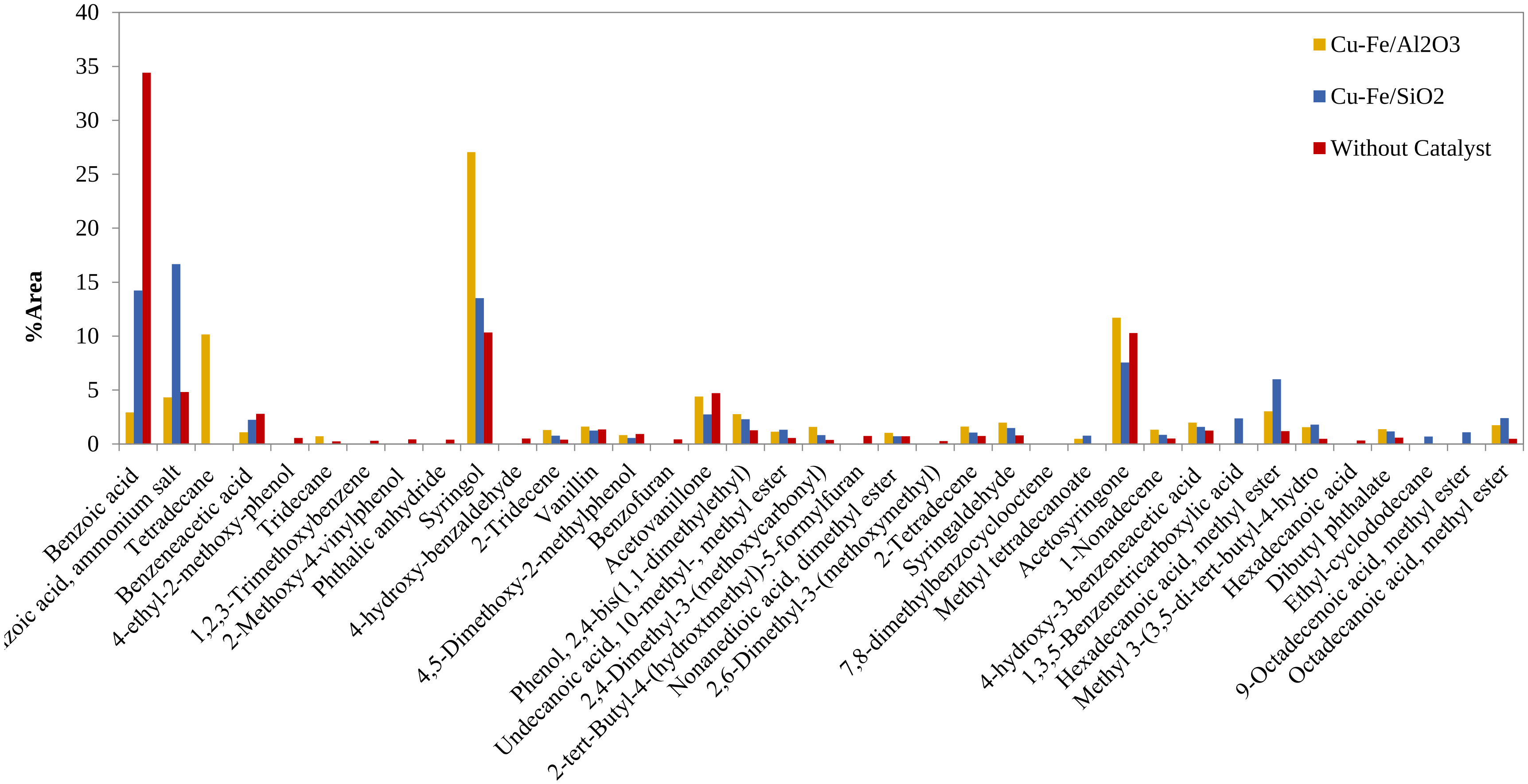

Figure S8 The phenolic compounds concentration peak area from GC-MS analysis for NaOH-lignin depolymerization with Cu-Fe/Al_2O_3, Cu-Fe/SiO_2, and without catalyst (microwave heating at 300 watts, 1%w/w of H_2O_2 in NaOH solution for 30 min).

Table S4 The phenolic compounds peak area percentage from GC-MS analysis for K_2CO_3-lignin depolymerization with Cu-Fe/Al_2O_3, Cu-Fe/SiO_2, and without catalyst (microwave heating at 300 watts, 1%w/w of H_2O_2 in NaOH solution for 15 min)

Cu-Fe/Al_2O_3			Cu-Fe/SiO_2			No catalyst		
RT	%Area	Type	RT	%Area	Type	RT	%Area	Type
		Benzoic acid				7.26	1.41	Benzoic acid
		Benzeneacetic acid, methyl ester	7.33	0.59	Benzeneacetic acid, methyl ester...	7.33	1.19	Benzeneacetic acid
7.38	3.32	1-methyl-2-Octyl-Cyclopropane	7.38	0.84		7.37	2.85	1-methyl-2-Octyl-Cyclopropane
		2-methyl-Benzaldehyde				7.64	0.42	Benzaldehyde, 2-methyl-
		2-Coumaranone				7.83	0.55	2-Coumaranone
		1,2,3-Trimethoxybenzene	8.32	0.32	1,2,3-Trimethoxybenzene	8.32	0.26	1,2,3-Trimethoxybenzene
		Heptanedioic acid, dimethyl ester				8.51	0.22	Heptanedioic acid, dimethyl ester
8.63	7.71	Syringol	8.63	6.94		8.63	4.89	
8.83	6.4	1-Tetradecene	8.83	4.18		8.83	5.18	
		3-(2-Hydroxyphenyl)propionic acid				8.93	0.42	3-(2-Hydroxyphenyl)propionic acid
		Vanillin				9	0.97	
9.58	0.63	Acetovanillone	9.58	1.08		9.58	2.44	
9.67	23.19	2,4-Di-tert-butylphenol	9.68	24.39	Phenol, 2,4-bis(1,1-dimethylethyl)	9.67	21.71	Phenol, 2,4-bis(1,1-dimethylethyl)
9.79	0.57	2-tert-Butyl-4-(hydroxtmethyl)-5	9.8	0.48				
		Nonanedioic acid, dimethyl ester	9.87	0.77	Nonanedioic acid, dimethyl ester			
10.17	9.03	1-Hexadecene	10.17	7.14		10.17	7.45	
10.21	0.32	Tetradecane				10.21	0.12	Tetradecane
		Diethylaminothyl Chloride Hydrochloride	10.56	0.39	Diethylaminothyl Chloride Hydrochloride			
10.66	0.41	Benzophenone	10.66	0.96		10.65	0.44	
11.05	0.47	1,2,3-trimethyl-4-p-Naphthalene,	11.06	0.47		10.97	0.24	
11.25	0.34	Methyl tetradecanoate	11.26	0.37				
11.31	0.55	1,1-Bis(p-tolyl)ethane	11.31	0.62		11.3	0.45	
11.42	0.36	Sodium-2,6-Diisopropylnaphthalene	11.43	0.44		11.42	0.32	
11.51	0.64	Acetosyringone	11.51	0.95		11.52	2.89	
11.96	7.26	1-Octadecene	11.97	7.74		11.95	6.07	
13.05	0.76	Bi-1,2-Benzenedicarboxylic acid	13.06	0.52				
13.81	8.56	Hexadecanoic acid, methyl ester	13.83	8.18		13.81	7.9	
14.27	7.56	Methyl-3-(3,5-Ditertbutyl-4-hydroxy)	14.29	10.55		14.26	5.27	
14.62	2.36	Dibutyl phthalate	14.64	2.62		14.62	2.09	
15.07	4.53	1-Nonadecene	15.08	4.74		15.06	4.32	
15.50	2.24	N-(2-(Diphenylphosphinyl)benzyl)	15.51	1.62		15.5	1.4	
17.82	0.76	9-Octadecenoic acid (Z)-, methyl	17.83	1.24		17.8	0.9	
18.63	4.91	Octadecanoic acid, methyl ester	18.66	5.94		18.63	5.39	
21.06	2.76	1-Chloro-hexadecane	21.1	2.02		21	2.19	
Total	95.64		Total	96.1		Total	89.95	

Table S5. The phenolic compounds peak area percentage from GC-MS analysis for K_2CO_3-lignin depolymerization with Cu-Fe/Al_2O_3, Cu-Fe/SiO_2, and without catalyst (microwave heating at 300 watts, 1%w/w of H_2O_2 in NaOH solution for 30 min)

Fe-Cu/Al_2O_3			Fe-Cu/SiO_2			Without Catalyst		
RT	%Area	Type	RT	%Area	Type	RT	%Area	Type
7.37	8.67	Benzoic acid, ammonium salt	7.37	9.09	Benzoic acid, ammonium salt	7.37	4.13	Benzoic acid, ammonium salt
7.43	2.65	Dodecace	7.43	12.04	Dodecace	7.43	12.59	Dodecace
		Benzoic acid				7.56	21.59	Benzoic acid
		2,6-Dimethylbenzaldehyde	7.69	0.32	2,6-Dimethylbenzaldehyde	7.69	12.79	2,6-Dimethylbenzaldehyde
		2-Coumaranone				7.83	1.09	2-Coumaranone
7.92	1.3	Benzeneacetic acid	7.92	2.04	Benzeneacetic acid	7.92		
		Acetic acid				8.01	6.19	Acetic acid
		Tridecane				8.18	0.78	Tridecane
8.32	0.28	1,2,3-Trimethoxybenzene	8.32			8.32	0.6	1,2,3-Trimethoxybenzene
		2-Methoxy-4-vinylphenol	8.38			8.38		
		1,3-Isobenzofurandione				8.46		
8.54	0.33	1,2-Dimethyl-4-(dimethoxymethyl)	8.54			8.54		
8.64	13.78	Syringol	8.64	10.07	Syringol	8.64	5.14	Syringol
		3-(2-Hydroxyphenyl)propionic acid				8.74		
8.83	5.03	1-Tetradecene	8.83	3.46	1-Tetradecene	8.83	0.94	1-Tetradecene
8.88	0.37	1,1'-Biphenyl	8.88	0.31		8.88		
9	0.45	Vanillin	9	0.41	Vanillin	9	1.19	Vanillin
		1-(4-hydroxyphenyl)-Ethanone				9.27	1.05	1-(4-hydroxyphenyl)-Ethanone
					Benzofuran	9.33		
9.58	2.13	Acetovanillone	9.58	1.6	Acetovanillone	9.58	5.27	Acetovanillone
9.67	20.98	2,4-Di-tert-butylphenol	9.67	20.02	2,4-Di-tert-butylphenol	9.67	2.76	2,4-Di-tert-butylphenol
		Dodecanoic acid, methyl ester				9.7	0.38	Dodecanoic acid, methyl ester
9.79	0.62	2-tert-Butyl-4-(hydroxtmethyl)	9.79	0.54	2-tert-Butyl-4-(hydroxtmethyl)	9.79		
10.17	8.21	1-Hexadecene	10.17	4.55	1-Hexadecene	10.17	1.35	1-Hexadecene
10.21	0.22	Hexadecane	10.21			10.21		
10.65	0.54	Benzophenone	10.65	0.47		10.65		
10.84	0.68	Syringaldehyde	10.84	0.87	Syringaldehyde	10.84	0.65	Syringaldehyde
11.05	0.43	1,2,3-trimethyl-4-p-Naphthalene	11.05	0.42	1,2,3-trimethyl-4-p-Naphthalene	11.05		
11.3	0.31	5,6-dihydro-5,6-dimethylbenzol	11.3	0.37	5,6-dihydro-5,6-dimethylbenzol	11.3		
11.42	0.29	2,6-Diisopropylnaphthalene	11.42	0.26	2,6-Diisopropylnaphthalene	11.42		
11.53	4.71	Acetosyringone	11.53	4.33	Acetosyringone	11.53	7.12	Acetosyringone
11.95	6.36	Heptadecenal	11.95	3.18	Heptadecenal	11.95	0.97	Heptadecenal
		4-hydroxy-3-Benzeneacetic acid	12.02	0.41	4-hydroxy-3-Benzeneacetic acid	12.02		
13.04	0.39	Bi-1,2-Benzenedicarboxylic acid	13.04	0.63	Bi-1,2-Benzenedicarboxylic acid	13.04		

Fe-Cu/Al_2O_3			Fe-Cu/SiO_2			Without Catalyst		
RT	%Area	Type	RT	%Area	Type	RT	%Area	Type
13.8	2.53	Hexadecanoic acid, methyl ester	13.8	5.77	Hexadecanoic acid, methyl ester	13.8	1.03	Hexadecanoic acid, methyl ester
14.25	2.07	Methyl 3-(3,5-di-tert-butyl-4-hydro)	14.25	4.78	Methyl 3-(3,5-di-tert-butyl-4-hydro)	14.25	0.88	Methyl 3-(3,5-di-tert-butyl-4-hydro)
14.62	2.26	Dibutyl phthalate	14.62	1.83	Dibutyl phthalate	14.62	0.97	Dibutyl phthalate
15.06	4.13	1-Octadecene	15.06	1.73	1-Octadecene	15.06	0.7	1-Octadecene
15.5	1.92	N-(2-(Diphenylphosphinyl)benzyl)	15.5	1.77	N-(2-(Diphenylphosphinyl)benzyl)	15.5	0.56	N-(2-(Diphenylphosphinyl)benzyl)
18.6	1.65	Octadecanoic acid, methyl ester	18.6	3.43	Octadecanoic acid, methyl ester	18.6	0.65	Octadecanoic acid, methyl ester
21.05	2.06	1-chloro-Hexadecane	21.05	0.85	1-chloro-Hexadecane	21.05		
Total	95.35		Total	95.55		Total	91.37	

Table S6. The phenolic compounds concentration peak area from GC-MS analysis for NaOH-lignin depolymerization with Cu-Fe/Al_2O_3, Cu-Fe/SiO_2, and without catalyst (microwave heating at 300 watts, 1%w/w of H_2O_2 in NaOH solution for 15 min).

Fe-Cu/Al_2O_3			Fe-Cu/SiO_2			Without Catalyst		
RT	%Area	Type	RT	%Area	Type	RT	%Area	Type
7.27	1.09	Benzoic acid				7.27	1.39	Benzoic acid
7.33	0.46	Benzeneacetic acid, methyl ester	7.33	4.08	Benzeneacetic acid, methyl ester	7.33		
7.37	2.87	Benzoic acid, ammonium salt		2.21	Benzoic acid, ammonium salt	7.37	10.47	Benzoic acid, ammonium salt
7.94		Acetic acid	7.94	2.71	Acetic acid	7.94	2.79	Acetic acid
8.03		3-methoxy- 1,2-benzenediol	8.03	1.15	3-methoxy- 1,2-benzenediol	8.03		
		7,7-dimethylbicyclo octane				8.11		
8.38	0.44	2-Methoxy-4-vinylphenol	8.38	0.4	2-Methoxy-4-vinylphenol	8.38	0.32	2-Methoxy-4-vinylphenol
8.64	13.39	Syringol	8.64	19.21	Syringol	8.64	5.86	Syringol
8.83	4.16	1-Tetradecene	8.83	2.77	1-Tetradecene	8.83	3.24	1-Tetradecene
9	1.39	Vanillin	9	2.156	Vanillin	9	1	Vanillin
9.09	0.8	4-Tricyclo decanone	9.09	1.1	4-Tricyclo decanone	9.09		
		Octanedioic acid, dimethyl ester				9.2	0.25	Octanedioic acid, dimethyl ester
9.58	2.33	Acetovanillone	9.58	3.69	Acetovanillone	9.58	2.84	Acetovanillone
9.67	20.17	2,4-Di-tert-butylphenol	9.67	27.29	2,4-Di-tert-butylphenol	9.67	13.09	2,4-Di-tert-butylphenol
9.79	0.84	2-tert-Butyl-4-(hydroxtmethyl)	9.79	1.23	2-tert-Butyl-4-(hydroxtmethyl)	9.79		2-tert-Butyl-4-(hydroxtmethyl)
10.16	6.53	1-Tetradecene	10.16	2.37	1-Tetradecene	10.16	4.62	1-Tetradecene
10.65	0.49	Benzophenone	10.65	0.71	Benzophenone	10.65	0.38	Benzophenone
10.84	0.98	Syringaldehyde	10.84	2.16	Syringaldehyde	10.84	0.77	Syringaldehyde
11.05	0.47	1,3-diformyl-2-chloro-5-isopropyl	11.05	0.48	1,3-diformyl-2-chloro-5-isopropyl	11.05	0.39	1,3-diformyl-2-chloro-5-isopropyl
11.3	0.45	1,2,3-trimethyl-4-p-Naphthalene	11.3	0.74	1,2,3-trimethyl-4-p-Naphthalene	11.3	0.32	1,2,3-trimethyl-4-p-Naphthalene
11.42	0.48	2,6-Diisopropylnaphthalene	11.42	0.42	2,6-Diisopropylnaphthalene	11.42	0.24	2,6-Diisopropylnaphthalene
11.53	4.72	Acetosyringone	11.53	9.36	Acetosyringone	11.53	6.39	Acetosyringone
11.95	5.47	Heptadecenal	11.95	1.07	Heptadecenal	11.95	4.03	Heptadecenal
12.02	0.68	4-hydroxy-3-benzeneacetic acid	12.02			12.02	1.13	4-hydroxy-3-benzeneacetic acid
13.04	0.54	Bi-1,2-Benzenedicarboxylic acid	13.04			13.04	0.35	Bi-1,2-Benzenedicarboxylic acid
13.8	6.91	Hexadecanoic acid, methyl ester	13.8	2.77	Hexadecanoic acid, methyl ester	13.8	5.44	Hexadecanoic acid, methyl ester
14.26	4.49	Methyl-3-(3,5-ditertbutyl-4-hydro-	14.26	1.88	Methyl-3-(3,5-ditertbutyl-4-hydro-	14.26	4.79	Methyl-3-(3,5-ditertbutyl-4-hydro-
14.61	1.81	Dibutyl phthalate	14.61	2.45	Dibutyl phthalate	14.61	1.25	Dibutyl phthalate
15.05	3	1,2-diethyl-cyclohexadecane	15.05			15.05	2.47	1,2-diethyl-cyclohexadecane
15.48	1.52	N-(2-(Diphenylphosphinyl)benzyl)	15.48	1.65	N-(2-(Diphenylphosphinyl)benzyl)	15.48	1.24	N-(2-(Diphenylphosphinyl)benzyl)
17.8	0.88	9-Octadecenoic acid, methyl ester	17.8	1.79	9-Octadecenoic acid, methyl ester	17.8	0.75	9-Octadecenoic acid, methyl ester
18.62	4.87	Octadecanoic acid, methyl ester	18.62			18.62	3.57	Octadecanoic acid, methyl ester
20.51	1.33	Lauramide	20.51			20.51	0.75	Lauramide
21.03	1.51	Trifluoroacetic acid, n-heptadec	21.03			21.03	1.37	Trifluoroacetic acid, n-heptadec
Total	95.07		Total	95.846		Total	81.5	

Table S7. The phenolic compounds concentration peak area from GC-MS analysis for NaOH-lignin depolymerization with Cu-Fe/Al_2O_3, Cu-Fe/SiO_2, and without catalyst (microwave heating at 300 watts, 1%w/w of H_2O_2 in NaOH solution for 30 min).

Fe-Cu/Al_2O_3			Fe-Cu/SiO_2			Without Catalyst		
RT	%Area	Type	RT	%Area	Type	RT	%Area	Type
7.33	2.92	Benzoic acid	7.33	14.24	Benzoic acid	7.33	34.42	Benzoic acid
7.38	4.32	Benzoic acid, ammonium salt	7.38	16.68	Benzoic acid, ammonium salt	7.38	4.823	Benzoic acid, ammonium salt
7.42	10.16	Tetradecane						
7.9	1.08	Benzeneacetic acid	7.9	2.25	Benzeneacetic acid	7.9	2.8	Benzeneacetic acid
		4-ethyl-2-methoxy-phenol				8.12	0.55	4-ethyl-2-methoxy-phenol
8.18	0.73	Tridecane				8.18	0.24	Tridecane
		1,2,3-Trimethoxybenzene				8.33	0.31	1,2,3-Trimethoxybenzene
		2-Methoxy-4-vinylphenol				8.38	0.42	2-Methoxy-4-vinylphenol
		Phthalic anhydride				8.47	0.41	Phthalic anhydride
8.64	27.06	Syringol	8.64	13.52	Syringol	8.64	10.34	Syringol
		4-hydroxy-benzaldehyde				8.75	0.5	4-hydroxy-benzaldehyde
8.82	1.29	2-Tridecene	8.82	0.76	2-Tridecene	8.82	0.41	2-Tridecene
9	1.61	Vanillin	9	1.25	Vanillin	9	1.35	Vanillin
9.26	0.81	4,5-Dimethoxy-2-methylphenol	9.26	0.56	4,5-Dimethoxy-2-methylphenol	9.26	0.94	4,5-Dimethoxy-2-methylphenol
		Benzofuran				9.34	0.44	Benzofuran
9.58	4.39	Acetovanillone	9.58	2.74	Acetovanillone	9.58	4.7	Acetovanillone
9.66	2.78	Phenol, 2,4-bis(1,1-dimethylethyl)	9.66	2.29	Phenol, 2,4-bis(1,1-dimethylethyl)	9.66	1.27	Phenol, 2,4-bis(1,1-dimethylethyl)
9.69	1.13	Undecanoic acid, 10-methyl-, methyl ester	9.69	1.33	Undecanoic acid, 10-methyl-, methyl ester	9.69	0.55	Undecanoic acid, 10-methyl-, methyl ester
9.79	1.58	2,4-Dimethyl-3-(methoxycarbonyl)	9.79	0.81	2,4-Dimethyl-3-(methoxycarbonyl)	9.79	0.37	2,4-Dimethyl-3-(methoxycarbonyl)
		2-tert-Butyl-4-(hydroxtmethyl)-5-formylfuran				9.8	0.75	2-tert-Butyl-4-(hydroxtmethyl)-5-formylfuran
9.86	1.03	Nonanedioic acid, dimethyl ester	9.86	0.73	Nonanedioic acid, dimethyl ester	9.86	0.72	
		2,6-Dimethyl-3-(methoxymethyl)				10.07	0.27	2,6-Dimethyl-3-(methoxymethyl)-p...
10.16	1.6	2-Tetradecene	10.16	1.05	2-Tetradecene	10.16	0.74	2-Tetradecene
10.84	1.97	Syringaldehyde	10.84	1.48	Syringaldehyde	10.84	0.79	Syringaldehyde
		7,8-dimethylbenzocyclooctene				11.11		7,8-dimethylbenzocyclooctene
11.24	0.48	Methyl tetradecanoate	11.24	0.78	Methyl tetradecanoate	11.24		
11.54	11.71	Acetosyringone	11.54	7.56	Acetosyringone	11.54	10.28	Acetosyringone
11.94	1.33	1-Nonadecene	11.94	0.84	1-Nonadecene	11.94	0.52	1-Nonadecene
12.02	1.99	4-hydroxy-3-benzeneacetic acid	12.02	1.58	4-hydroxy-3-benzeneacetic acid	12.02	1.23	4-hydroxy-3-benzeneacetic acid
		1,3,5-Benzenetricarboxylic acid	12.6	2.37	1,3,5-Benzenetricarboxylic acid			
13.79	3.03	Hexadecanoic acid, methyl ester	13.79	5.99	Hexadecanoic acid, methyl ester	13.79	1.18	Hexadecanoic acid, methyl ester
14.23	1.56	Methyl 3-(3,5-di-tert-butyl-4-hydro	14.23	1.8	Methyl 3-(3,5-di-tert-butyl-4-hydro	14.23	0.49	Methyl 3-(3,5-di-tert-butyl-4-hydro
		Hexadecanoic acid				14.44	0.32	Hexadecanoic acid
14.6	1.37	Dibutyl phthalate	14.6	1.17	Dibutyl phthalate	14.6	0.58	Dibutyl phthalate
15.04		Ethyl-cyclododecane	15.04	0.69	Ethyl-cyclododecane			
17.81		9-Octadecenoic acid, methyl ester	17.81	1.08	9-Octadecenoic acid, methyl ester			
18.59	1.73	Octadecanoic acid, methyl ester	18.59	2.4	Octadecanoic acid, methyl ester	18.59	0.48	Octadecanoic acid, methyl ester
Total	87.66		Total	85.95		Total	83.193	

Table S8. Recyclability study of $CuFe/Al_2O_3$ and $CuFe/SiO_2$ catalysts on depolymerization of NaOH-lignin under microwave at 300W for 30 min.

$CuFe/Al_2O_3$ catalyst						
Products	1st reaction		2nd reaction		3rd reaction	
	Concentration (mg L^{-1})	Concentration (wt%)	Concentration (mg L^{-1})	Concentration (wt%)	Concentration (mg L^{-1})	Concentration (wt%)
S1 (Syringol)	2.82	4.50	5.25	10.10	2.01	5.19
S2 (Syringaldehyde)	38.46	61.31	22.92	44.11	14.75	38.05
S3 (Acetosyringone)	9.46	15.08	6.48	12.46	11.36	29.29
G1 (Vanillin)	4.98	7.95	6.32	12.17	6.81	17.58
G2 (Acetovanillone)	4.11	6.55	3.14	6.05	3.83	9.88
H1 (2,4-Di-tert butylphenol)	2.89	4.61	7.84	15.09	0.00	0.00
Total phenolic products	62.73	100.00	51.95	100.00	38.76	100.00
$CuFe/SiO_2$ catalyst						
Products	1st reaction		2nd reaction		3rd reaction	
	Concentration (mg L^{-1})	Concentration (wt%)	Concentration (mg L^{-1})	Concentration (wt%)	Concentration (mg L^{-1})	Concentration (wt%)
S1 (Syringol)	2.26	4.21	4.66	12.47	2.70	23.12
S2 (Syringaldehyde)	17.68	32.95	21.17	56.68	0.00	0.00
S3 (Acetosyringone)	20.06	37.37	3.81	10.20	3.02	25.81
G1 (Vanillin)	5.23	9.74	4.88	13.05	3.66	31.28
G2 (Acetovanillone)	3.01	5.62	2.84	7.60	2.31	19.78
H1 (2,4-Di-tert butylphenol)	5.42	10.10	0.00	0.00	0.00	0.00
Total phenolic products	53.66	100.00	37.35	100.00	11.69	100.00

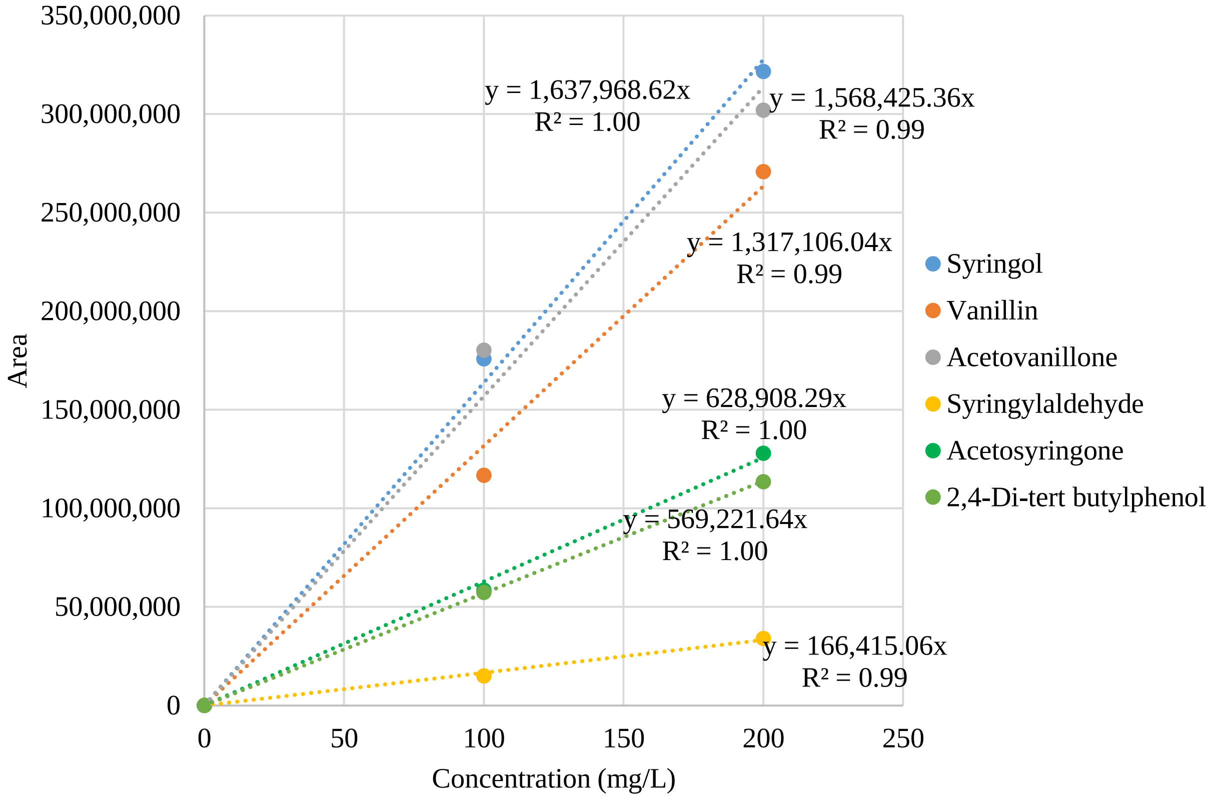

Figure S9 Standard curve from GC analysis of main products of lignin depolymerization for recyclability study.
